# Systematic Pan-Cancer Analysis Identifies TREM2 as an Immunological and Prognostic Biomarker

**DOI:** 10.3389/fimmu.2021.646523

**Published:** 2021-02-17

**Authors:** Xin Cheng, Xiaowei Wang, Kechao Nie, Lin Cheng, Zheyu Zhang, Yang Hu, Weijun Peng

**Affiliations:** ^1^Department of Integrated Traditional Chinese & Western Medicine, The Second Xiangya Hospital, Central South University, Changsha, China; ^2^Department of Pathology, The Second Xiangya Hospital, Central South University, Changsha, China

**Keywords:** TREM2, pan-cancer, prognosis, immune infiltration, TMB, MSI

## Abstract

Triggering receptor expressed on myeloid cells-2 (TREM2) is a transmembrane receptor of the immunoglobulin superfamily and a crucial signaling hub for multiple pathological pathways that mediate immunity. Although increasing evidence supports a vital role for TREM2 in tumorigenesis of some cancers, no systematic pan-cancer analysis of TREM2 is available. Thus, we aimed to explore the prognostic value, and investigate the potential immunological functions, of TREM2 across 33 cancer types. Based on datasets from The Cancer Genome Atlas, and the Cancer Cell Line Encyclopedia, Genotype Tissue-Expression, cBioPortal, and Human Protein Atlas, we employed an array of bioinformatics methods to explore the potential oncogenic roles of TREM2, including analyzing the relationship between TREM2 and prognosis, tumor mutational burden (TMB), microsatellite instability (MSI), DNA methylation, and immune cell infiltration of different tumors. The results show that TREM2 is highly expressed in most cancers, but present at low levels in lung cancer. Further, TREM2 is positively or negatively associated with prognosis in different cancers. Additionally, TREM2 expression was associated with TMB and MSI in 12 cancer types, while in 20 types of cancer, there was a correlation between TREM2 expression and DNA methylation. Six tumors, including breast invasive carcinoma, cervical squamous cell carcinoma and endocervical adenocarcinoma, kidney renal clear cell carcinoma, lung squamous cell carcinoma, skin cutaneous melanoma, and stomach adenocarcinoma, were screened out for further study, which demonstrated that TREM2 gene expression was negatively correlated with infiltration levels of most immune cells, but positively correlated with infiltration levels of M1 and M2 macrophages. Moreover, correlation with TREM2 expression differed according to T cell subtype. Our study reveals that TREM2 can function as a prognostic marker in various malignant tumors because of its role in tumorigenesis and tumor immunity.

## Introduction

Cancer is a leading cause of death and major obstacle affecting the quality of life in every country globally, and to date, there are no absolute cures for cancer (1). In recent years, cancer immunotherapy has become a prominent cancer treatment, especially immune checkpoint blocking therapy (2). With the continuous development and improvement of public databases such as The Cancer Genome Atlas (TCGA), it is possible to discover new immunotherapy targets by performing pan-cancer expression analysis of genes and evaluating their correlations with clinical prognosis and related signaling pathways (3).

Triggering receptor expressed on myeloid cell 2 (TREM2) is a transmembrane receptor of the immunoglobulin superfamily, which can inhibit the phagocytic function of dendritic cells and macrophages, thereby affecting related immune signaling pathways (4). TREM2 has vital roles in Alzheimer's disease and other neurodegenerative diseases, and is involved in numerous immune and inflammatory pathways that contribute to the etiology of these diseases (5, 6). Recently, accumulating evidence has suggested that TREM2 also has an impact on the occurrence and development of tumors. TREM2 affects the prognosis and clinical phenotype of tumors through its role in tumor-associated macrophages (TAMs) and myeloid-derived suppressor cells (MDSCs) (7, 8). TREM2 can alter the morphology of tumor-infiltrating macrophages, inhibit tumor growth, and enhance checkpoint blocking therapy (9).

Furthermore, recent scRNA-seq studies of the tumor microenvironment (TME) have revealed that TREM2 is expressed in several subgroups of myeloid cells, some of which overlap with MDSCs according to various definitions (10). TREM2 is generally considered to be an anti-tumor factor, acting through the Sky and Wnt1/β-catenin pathways in multiple cancers, including hepatocellular carcinoma (11), colorectal cancer (12), and lung cancer (13); however, TREM2 is expressed at higher levels in gastric cancer (14), and its up-regulation is related to poor patient prognosis. Similar results have been reported in renal cell carcinoma (15). In summary, increasing evidence suggests that TREM2 is an emerging immunosuppressor in the TME, and that its expression can impact patient prognosis.

Nevertheless, most research studies into the role of TREM2 in tumors to date have been limited to a specific type of cancer. There has been no pan-cancer study of the association between TREM2 and various cancers. Therefore, we used multiple databases, including TCGA, Cancer Cell Line Encyclopedia (CCLE), Genotype Tissue-Expression (GTEx), cBioPortal, and Human Protein Atlas (HPA), to analyze TREM2 expression levels and their relationship with prognosis in different types of malignancy. We also explored the potential associations between TREM2 expression and microsatellite instability (MSI), tumor mutational burden (TMB), DNA methylation, and immune infiltration levels across 33 types of cancer. Further, we conducted co-expression analyses of immune-related and mismatch repair (MMR) genes with TREM2 and enrichment analysis to study the biological functions of TREM2 in tumors. Our results show that TREM2 can be used as a prognostic factor for a variety of cancers, and TREM2 can play an important role in tumor immunity by affecting tumor infiltrating immune cells, TMB, and MSI. This study can provide insight into the role of TREM2 in tumor immunotherapy.

## Methods

### Data Processing and Differential Expression Analysis

RNA sequencing, somatic mutation, and related clinical data were downloaded from TCGA (contains 11069 samples from 33 types of cancer) using UCSC Xena (https://xena.ucsc.edu/), an online tool for exploration of gene expression, clinical, and phenotype data. Data from each tumor cell line downloaded from the CCLE database (https://portals.broadinstitute.org/ccle/) and expression levels in 21 tissues were analyzed according to the tissue source. Gene expression data from 31 different tissues were downloaded from GTEx (https://commonfund.nih.gov/GTEx). Strawberry Perl (Version 5.32.0, http://strawberryperl.com/) was used to extract TREM2 gene expression data from these downloaded data sets and plot it into a data matrix for subsequent analyses.

The expression of TREM2 was evaluated in 31 normal tissues, each of 21 tumor cell lines, and 33 tumors, using the downloaded data and expression levels compared between cancer samples and matched standard samples in 33 cancers. Expression data were Log2 transformed and two sets of *t*-tests conducted on these tumor types; *P* < 0.05 were considered to indicate differential expression between tumor and normal tissues. Data analysis was conducted using R software (Version 4.0.2; https://www.R-project.org), and the R package “ggpubr” used to draw box plots.

### Immunohistochemistry (IHC) Staining

To evaluate differences in TREM2 expression at the protein level, IHC images of TREM2 protein expression in normal tissues and seven tumors tissues, including liver hepatocellular carcinoma (LIHC), colon adenocarcinoma (COAD), head and neck squamous cell carcinoma (HNSC), cervical squamous cell carcinoma and endocervical adenocarcinoma (CESC), breast invasive carcinoma (BRCA), lung squamous cell carcinoma (LUSC), lung adenocarcinoma (LUAD), were downloaded from the HPA (http://www.proteinatlas.org/) and analyzed.

### Analysis of the Relationships Between TREM2, Prognosis, and Clinical Phenotype

Survival and clinical phenotype data were extracted for each sample downloaded from TCGA. Four indicators, overall survival (OS), disease-specific survival (DSS), disease-free interval (DFI), and progression-free interval (PFI), were selected to study the relationship between TREM2 expression and patient prognosis. The Kaplan-Meier method and log-rank test were used for survival analyses (*p* < 0.05) of each cancer type. Survival curves were drawn using the R packages “survival” and “survminer.” Moreover, Cox analysis was conducted using the R packages “survival” and “forestplot” to determine the pan-cancer relationship between TREM2 expression and survival.

Two clinical phenotypes, tumor stage and patient age, were selected and their relationship with TREM2 expression explored. Patients were divided into two groups, with 65 years old as the cutoff value. Clinical phenotype correlation analyses were conducted using the R-packages “limma” and “ggpubr”; *p* < 0.05 were considered significant.

### Correlation of TREM2 Expression With Tumor Mutation Burden, Tumor Microsatellite Instability, and Mismatch Repair Gene Expression

TMB is a quantifiable immune-response biomarker that reflects the number of mutations in tumor cells (16). MSI results from MMR deficiency and is associated with patient outcomes (17). TMB scores were calculated using a Perl script and corrected by dividing by the total length of exons. MSI scores were determined for all samples based on somatic mutation data downloaded from TCGA (https://tcga.xenahubs.net) and the relationship between TREM2 expression and TMB and MSI analyzed using Spearman's rank correlation coefficient. Results are presented as heatmap, generated using the R-package “reshape2” and “RColorBrewer.” MMR is a DNA repair mechanism in cells. Down-regulation or functional defects in MMR genes lead to DNA replication errors that cannot be repaired, resulting in higher frequencies of somatic mutations (18). Expression profile data from TCGA were used to evaluate the levels of the MMR genes, MutL homologous gene (MLH1), MutS homologous gene (MSH2), MSH6, increased separation after meiosis (PMS2), epithelial cell adhesion molecule (EPCAM), in different cancers and determine the correlation between levels of MMR gene expression and that of TREM2. Data were visualized as heat maps generated using the R-packages, “reshape2” and “RColorBrewer.”

### Relationship Between TREM2 Expression and Immunity

Estimation of Stromal and Immune Cells in Malignant Tumor Tissues Using Expression Data (ESTIMATE) is a method for inferring the degree of infiltration of stromal or immune cells into tumors using existing gene expression profiles (19). The ESTIMATE algorithm was used to calculate immune and stromal scores for each tumor sample and the relationship between TREM2 expression and these two scores evaluated according to the degree of immune infiltration using the R software packages “estimate” and “limma.”

Moreover, we used CIBERSORT, a metagene tool which can predict the phenotypes of immunocytes, to calculate relative scores for 26 immune cells in 32 cancers [except acute myeloid leukemia (LAML)] (20). Correlations between TREM2 levels and each immune cell infiltration level in cancer were evaluated using the R-packages “ggplot2”, “ggpubr,” and “ggExtra” (*P* < 0.05 as significant).

In addition, we conducted a co-expression analysis of TREM2 and immune-related genes, including genes encoding major histocompatibility complex (MHC), immune activation, immunosuppressive, chemokine, and chemokine receptor proteins, using the R-package “limma”; the “reshape2” and “RColorBreyer” packages were used to visualize the results.

### Correlation of TREM2 Expression With DNA Methylation

DNA methylation is a form of DNA chemical modification, and as an essential regulator of gene transcription, can be carcinogenic (21). HM450 methylation data from cBioPortal (www.cbioportal.org) were used. Analysis of the correlation between TREM2 expression and gene promoter methylation was conducted for each tumor. Correlation of TREM2 methylation with prognosis was conducted using Kaplan-Meier survival analysis, including OS, DSS, DFI, and PFI (*P* < 0.05 as significant).

### The Biological Significance of TREM2 Expression in Tumors

Gene Set Enrichment Analysis (GSEA) and the Gene Set Variation Analysis (GSVA) were conducted to investigate the biological functions of TREM2 in tumors. Gene ontology (GO) and Kyoto Encyclopedia of Genes and Genomes (KEGG) gene sets were downloaded from the official GSEA website (https://www.gsea-msigdb.org/gsea/downloads.jsp). Functional analysis was performed using the R-packages “limma,” “org.Hs.eg.db,” “clusterProfiler,” and “enrichplot.” The GSVA gene set was from the MSigDB database (v7.2 updated September 2020; https://www.gsea-msigdb.org/gsea/msigdb/index.jsp). GSVA scores were generated for all tumors, and then samples in each tumor divided into two groups with high and low expression, using the median of differential genes with the R-package “limma.” Correlation of TREM2 expression with more than 20,000 pathways in each tumor was analyzed and the 15 pathways with the most significant positive and negative correlations visualized.

### Statistical Analysis

All the data of gene expression was normalized by log2 transformation. Comparison of normal tissue and cancer tissue used two sets of *t*-test; *P* < 0.05 was indicated the statistical significance. The Kaplan-Meier curve, log-rank test and Cox proportional hazard regression model were used for all survival analyses in this study. The correlation analysis between the two variables used Spearman's or Pearson's test; *P* < 0.05 were considered significant. All statistical analyses were processed by the R software (Version 4.0.2).

## Results

### Differential Expression of TREM2 Between Tumor and Normal Tissue Samples

**Figure 1:**
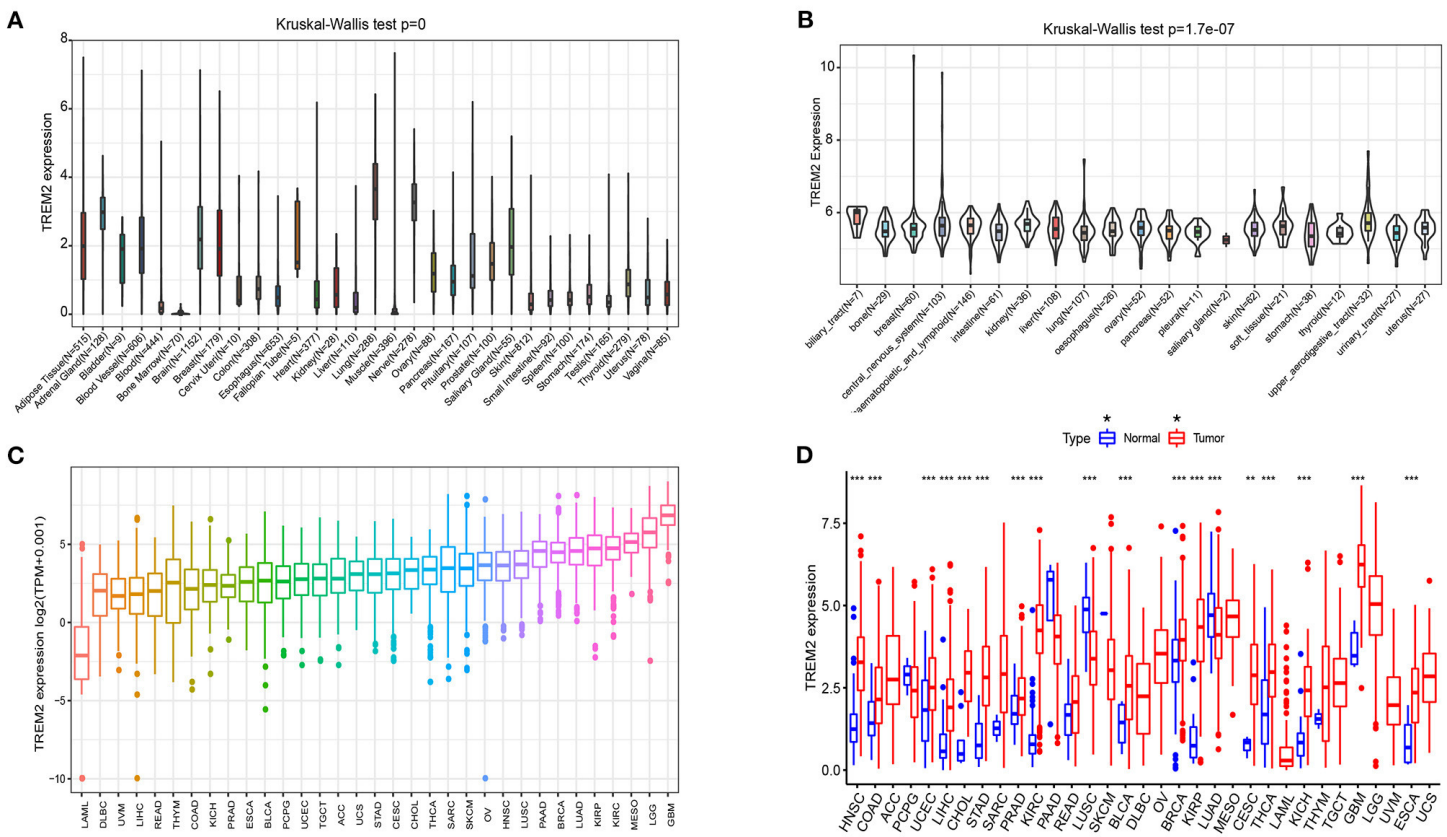
Differential expression of TREM2. **(A)** TREM2 expression in normal tissues. **(B)** TREM2 expression in tumor cell lines. **(C)** TREM2 expression in 33 types of cancer. **(D)** Comparison of TREM2 expression between tumor and normal samples. *P < 0.05, **P < 0.01, ***P < 0.001.

We analyzed physiologic TREM2 gene expression levels across tissues, using the GTEx data set (Figure 1A). Expression levels were highest in lung tissue, while most other normal tissues expressed low levels of TREM2. Relative TREM2 expression levels across different cell lines from CCLE data are presented in Figure 1B. In most normal cells, TREM2 expression levels were relatively low (*p* < 0.001), consistent with the results of analysis of GTEx data.

Next, we analyzed TREM2 expression levels in various cancers and ranked them from low to high (Figure 1C). All cancers expressed TREM2, with the highest levels in glioblastoma multiforme (GBM) and the lowest in LAML. We also compared TREM2 expression levels between cancer and matched normal samples from 33 cancers, based on TCGA data (Figure 1D). Except for those cancers where no normal tissue data was available, significant differences in TREM2 expression were detected between tumor and normal tissue in 18 types of cancer. Among them, TREM2 was highly expressed in HNSC, COAD, uterine corpus endometrial carcinoma, LIHC, cholangiocarcinoma (CHOL), stomach adenocarcinoma (STAD), prostate adenocarcinoma (PRAD), kidney renal clear cell carcinoma (KIRC), bladder urothelial carcinoma (BLCA), breast invasive carcinoma (BRCA), kidney renal papillary cell carcinoma (KIRP), CESC, thyroid carcinoma (THCA), kidney chromophobe (KICH), GBM, and esophageal carcinoma (ESCA). In contrast, TREM2 levels were downregulated in tumor relative to normal tissues in LUSC and LUAD. Notably the largest difference between expression of TREM2 in cancer and normal tissues was for KIRP and KIRC; however, there was no significant difference in TREM2 levels between rectum adenocarcinoma (READ) and non-tumor tissues. Some cancers only had very few normal samples (for example, there were only data from two normal tissue samples in the sarcoma (SARC) dataset) and differences were not significant, likely because of the small number of samples.

**Figure 2:**
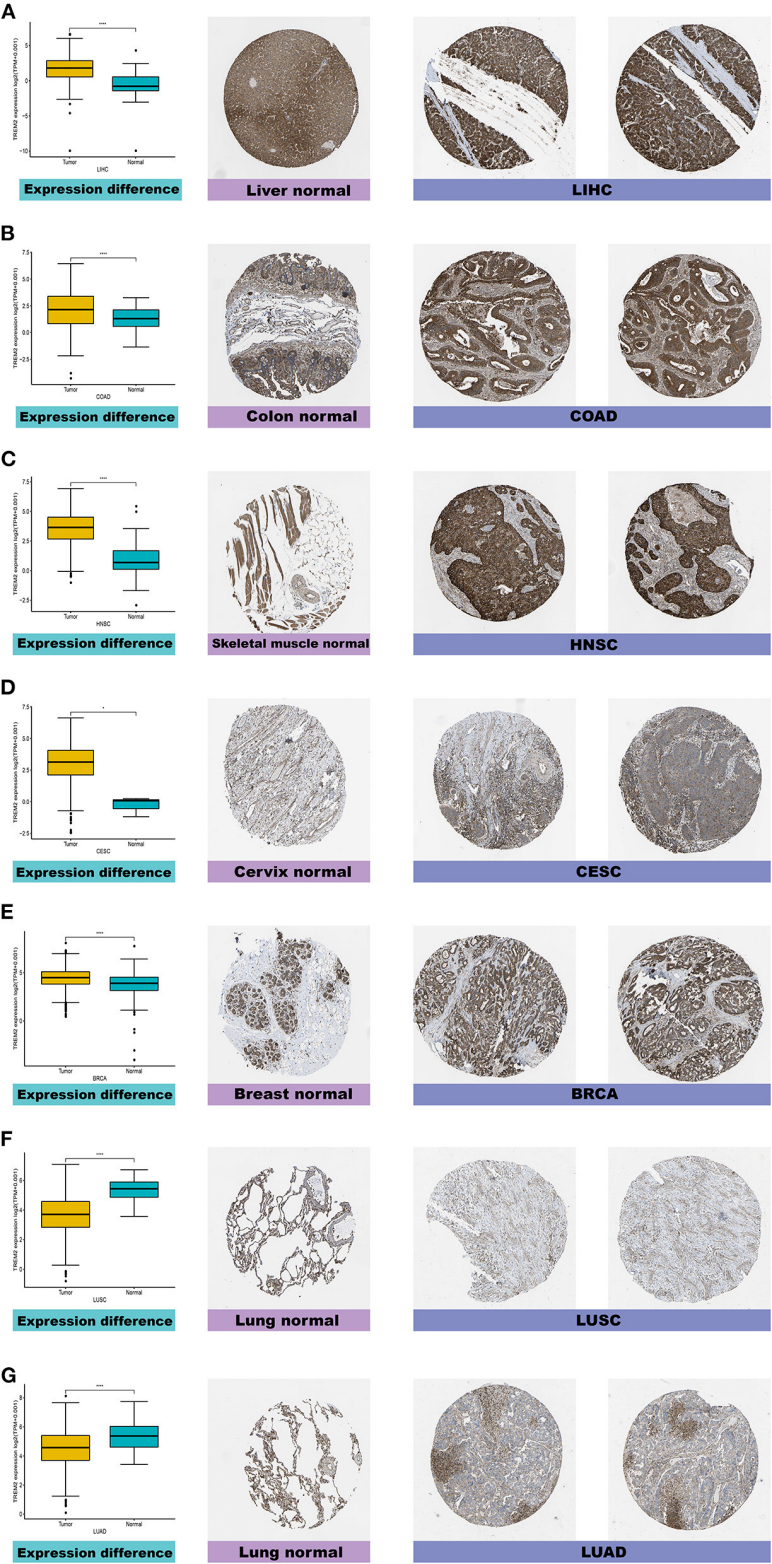
Comparison of TREM2 gene expression between normal and tumor tissues (left) and immunohistochemistry images in normal (middle) and tumor (right) tissues. TREM2 protein expression was significantly higher in liver hepatocellular carcinoma (LIHC), colon adenocarcinoma (COAD), head and neck squamous cell carcinoma (HNSC), cervical squamous cell carcinoma and endocervical adenocarcinoma (CESC), and breast invasive carcinoma (BRCA) tissues than normal tissues. **(A)** Liver. **(B)** Colon. **(C)** Skeletal muscle. **(D)** Cervix. **(E)** Breast. **(F,G)** Lung. ****P < 0.0001.

Furthermore, to evaluate TREM2 expression at the protein level, we analyzed IHC results provided by the HPA database and compared the results with TREM2 gene expression data from TCGA. As shown in Figures 2A–G, the results of analysis of data from these two databases were consistent with one another. Normal liver, colon, skeletal muscle, and breast tissues had moderate TREM2 IHC staining, while tumor tissues had strong staining. Normal cervix tissue samples had weak TREM2 staining, while tumor tissues had moderate staining. Conversely, normal lung tissues had moderate TREM2 staining, while LUSC and LUAD had weak or no TREM2 staining.

### Prognostic Value of TREM2 Across Cancers

**Figure 3:**
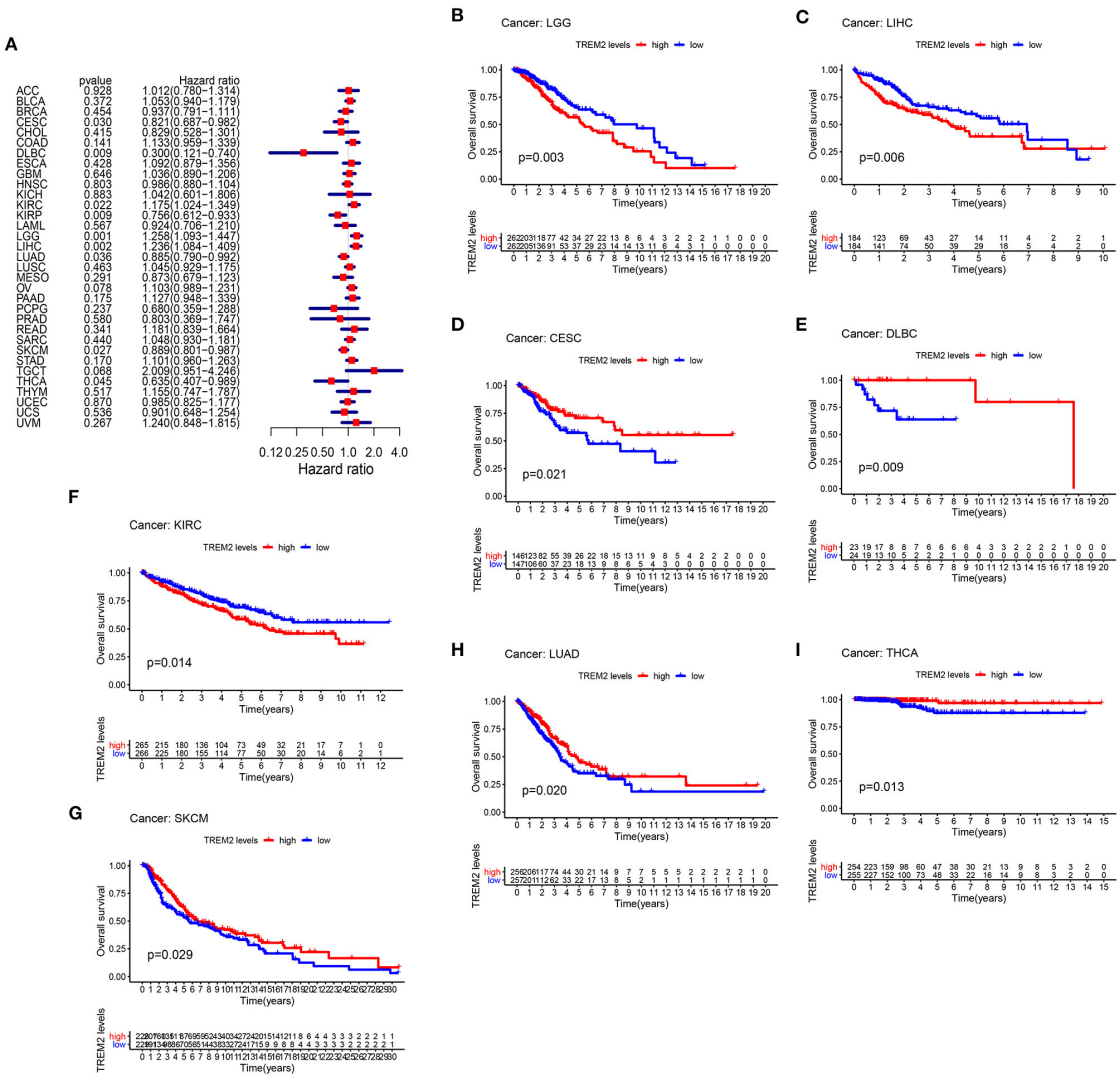
Association between TREM2 expression and overall survival time in days (OS). **(A)** Forest plot of OS associations in 33 types of tumor. **(B–I)** Kaplan-Meier analysis of the association between TREM2 expression and OS.

To study the association between TREM2 expression level and prognosis, we performed a survival association analysis for each cancer, including OS, DSS, DFI, and PFI. Cox proportional hazards model analysis showed that TREM2 expression levels were associated with OS in CESC (*p* = 0.030), lymphoid neoplasm diffuse large B-cell lymphoma (DLBC) (*p* = 0.009), KIRC (*p* = 0.022), KIRP (*p* = 0.009), brain lower grade glioma (LGG) (*p* = 0.001), LIHC (*p* = 0.002), LUAD (*p* = 0.036), skin cutaneous melanoma (SKCM) (*p* = 0.027), and THCA (*p* = 0.045) (Figure 3A). Further, TREM2 was a high-risk gene in KIRC, LGG, and LIHC, while it was a low-risk gene in other types of cancer, particularly DBLC (hazard ratio = 0.3000). Kaplan-Meier survival analysis also demonstrated that among patients with CESC (Figure 3D; *p* = 0.021), DLBC (Figure 3E; *p* = 0.009), LUAD (Figure 3H; *p* = 0.020), THCA (Figure 3I; *p* = 0.013), and SKCM (Figure 3G, *p* = 0.029), those with high levels of TREM2 had longer survival times, while in patients with LGG (Figure 3B; *P* = 0.003), LIHC (Figure 3C; *p* = 0.006), and KIRC (Figure 3F; *p* = 0.014), high TREM2 expression was associated with poor OS.

**Figure 4:**
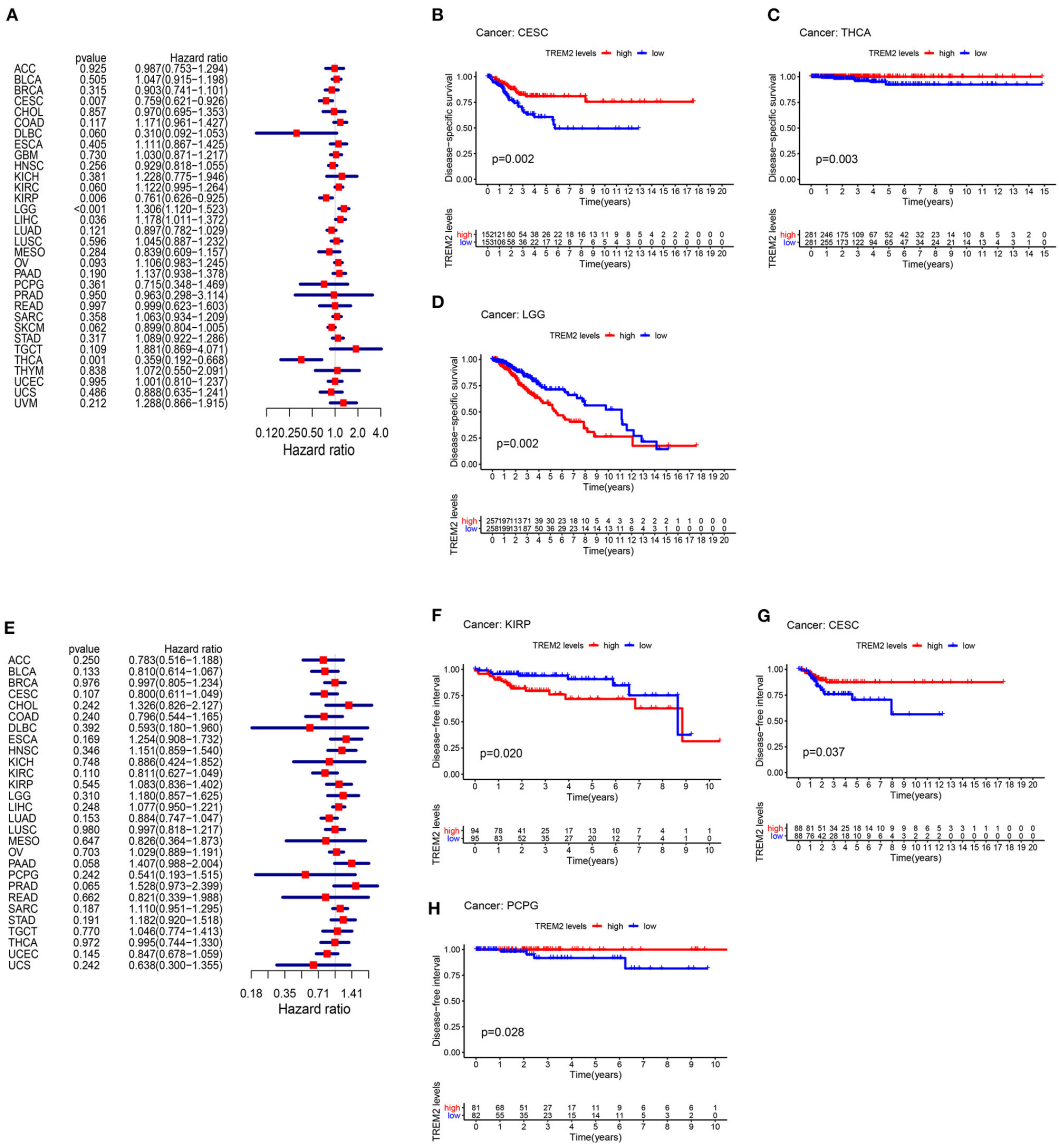
Association between TREM2 expression levels and disease-specific survival (DSS) and disease-free interval (DFI). **(A)** Forest plot of association of TREM2 expression and DSS in 33 types of tumor. **(B–D)** Kaplan-Meier analysis of the association between TREM2 expression and DSS. **(E)** Forest plot of association of TREM2 with DFI for 33 types of tumor. **(F–H)** Kaplan-Meier analysis of the association between TREM2 expression and DFI.

**Figure 5:**
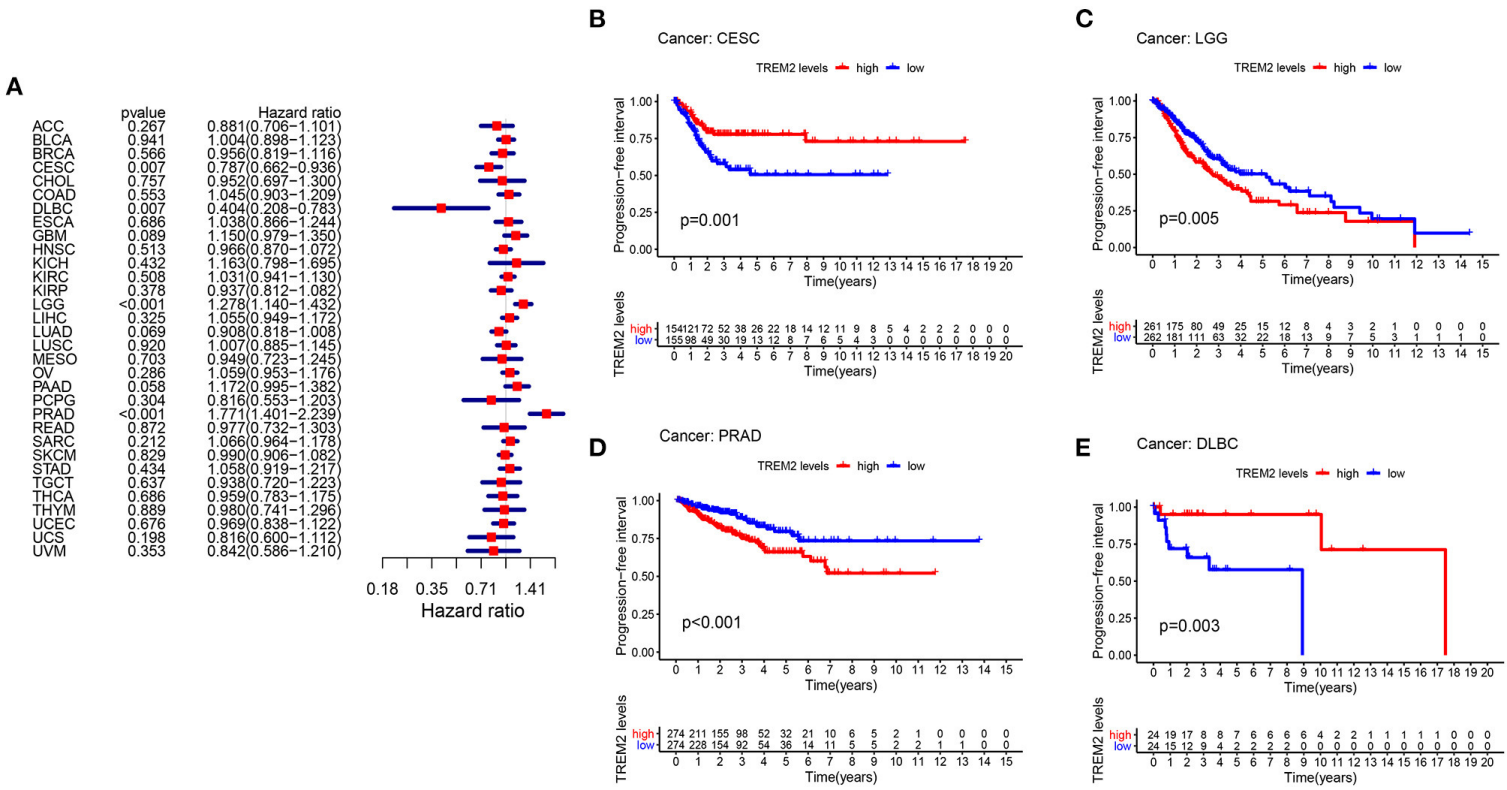
Association between TREM2 expression and progression-free interval (PFI). **(A)** Forest plot of PFI association with TREM2 expression in 33 tumor types. **(B–E)** Kaplan-Meier analysis of the association between TREM2 expression and PFI.

Moreover, analysis of DSS data (Figure 4A) revealed associations between low TREM2 expression and poor prognosis in patients with CESC (*P* = 0.007), KIRP (*p* = 0.006), and THCA (*p* = 0.001); however, in patients with LGG (*p* < 0.001) and LIHC (*p* = 0.036), TREM2 expression exhibited the opposite relationship with prognosis. Kaplan-Meier survival analysis revealed a correlation between TREM2 expression level and poor prognosis in patients with CESC (Figure 4B; *p* = 0.002), THCA (Figure 4C; *p* = 0.003), and LGG (Figure 4D; *p* = 0.002). No correlation was detected between TREM2 expression and DFI in any type of cancer (Figure 4E; all *p* > 0.05); however, significant relationships were detected in KIRP (Figure 4F; *p* = 0.020), CESC (Figure 4G; *p* = 0.037), and PCPG (Figure 4H; *p* = 0.028) by KM survival analysis. Regarding associations between TREM2 expression and PFI, forest plots showed associations between high expression and poor PFI in LGG (*p* < 0.001) and PRAD (*p* < 0.001), while low expression was associated with poor PFI in patients with CESC (*p* = 0.007) and DLBC (*p* = 0.007) (Figure 5A). KM analysis showed that individuals with in CESC (Figure 5B; *p* = 0.001) and DLBC (Figure 5E; *p* = 0.003) and high levels of TREM2 expression had longer survival times, while patients with LGG (Figure 5C; *p* = 0.005) and PRAD (Figure 5D; *p* < 0.001) and high TREM2 expression had poor PFI.

### Correlation of TREM2 Expression With Clinical Phenotypes in Various Cancers

**Figure 6:**
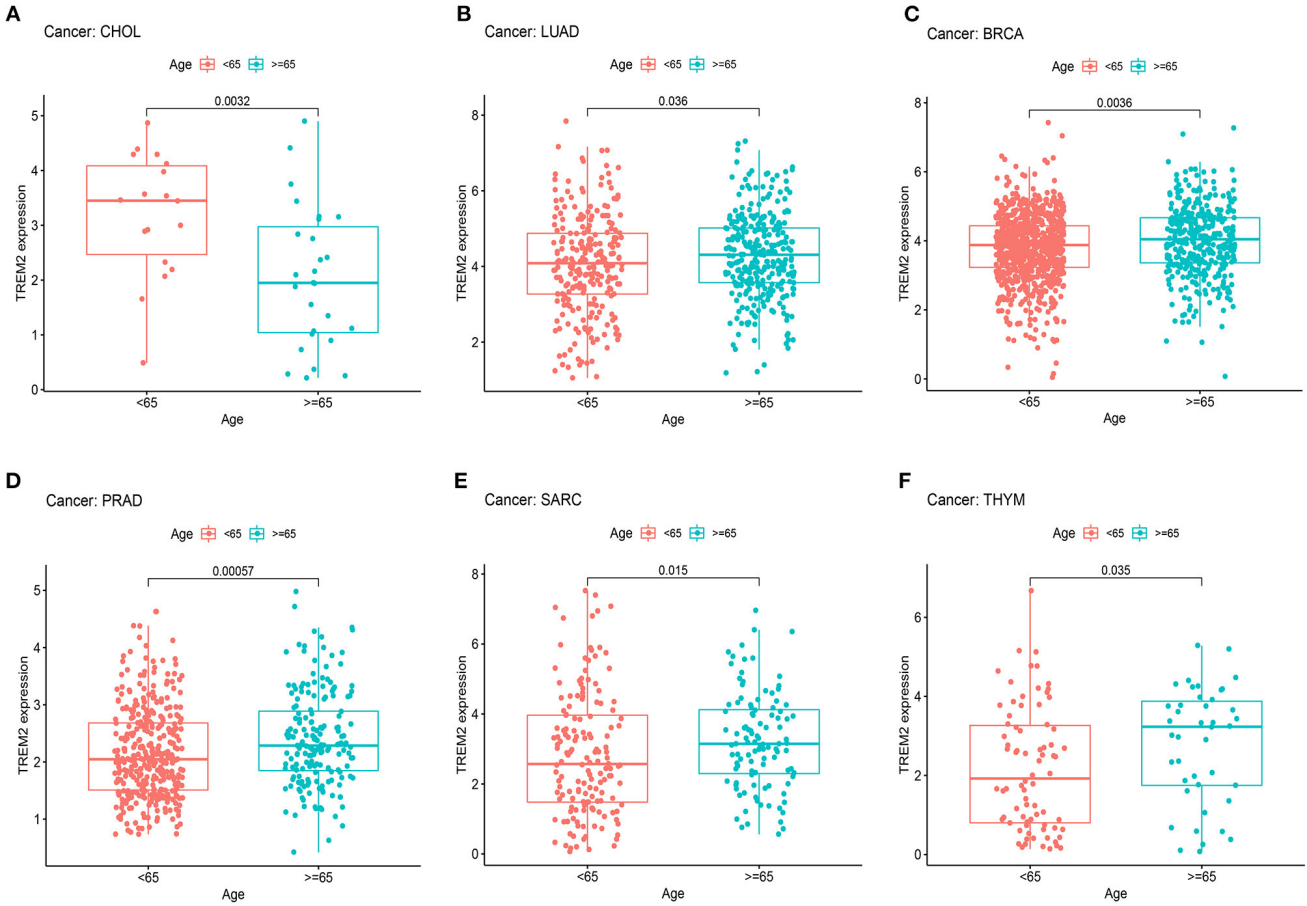
Association between TREM2 expression and age in **(A)** cholangiocarcinoma (CHOL), **(B)** lung adenocarcinoma (LUAD), **(C)** breast invasive carcinoma (BRCA), **(D)** prostate adenocarcinoma (PRAD), **(E)** sarcoma (SARC), and **(F)** thymoma (THYM).

Next, we examined the differential expression of TREM2 according to age for patients with each tumor type and found that those aged ≥ 65 years with LUAD (Figure 6B; *p* = 0.036), BRCA (Figure 6C; *p* = 0.0036), PRAD (Figure 6D; *p* = 0.0006), SARC (Figure 6E; *p* = 0.015), and thymoma (THYM) (Figure 6F; *p* = 0.035) had higher expression levels, while patients with CHOL < 65 years had higher TREM2 expression levels (Figure 6A; *p* = 0.0032). No significant correlations between age and TREM2 expression were detected in patients with other cancers.

**Figure 7:**
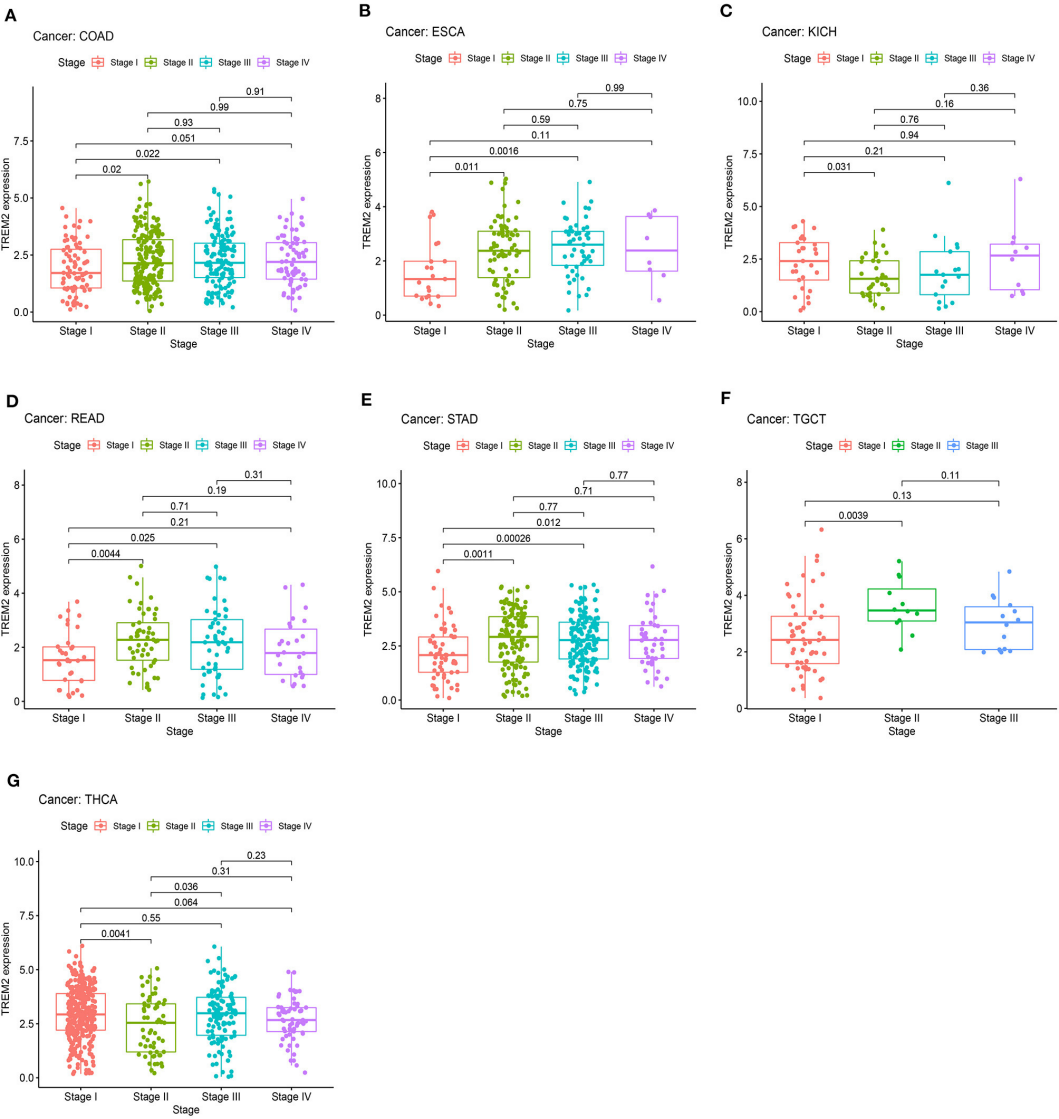
Association between TREM2 expression and tumor stage in **(A)** colon adenocarcinoma (COAD), **(B)** esophageal carcinoma (ESCA), **(C)** kidney chromophobe (KICH), **(D)** rectum adenocarcinoma (READ), **(E)** stomach adenocarcinoma (STAD), **(F)** testicular germ cell tumors (TGCT), and **(G)** thyroid carcinoma (THCA).

We also analyzed the relevance of tumor stage, and found that TREM2 expression significantly correlated with tumor stage in thirteen types of cancer, including BLCA, BRCA, COAD, ESCA, KICH, KIRC, KIRP, LUAD, mesothelioma (MESO), READ, STAD, testicular germ cell tumors (TGCT), and THCA (Supplementary Figure 1). Notably, the majority of significant differences in TREM2 expression were between stage I and II tumors (Figure 7). Interestingly, as shown in Figure 7, TREM2 expression increased from stage I to stage II, other than in patients with KICH (Figure 7C; *p* = 0.031) and THCA (Figure 7G; *p* = 0.0041). Although the difference between stage I and II tumors was significant, differences between higher stage tumors were relatively small and not statistically significant in most types of cancer.

### Correlations of TREM2 Expression Levels With Tumor Mutation Burden, Tumor Microsatellite Instability, and Mismatch Repair Genes

**Figure 8:**
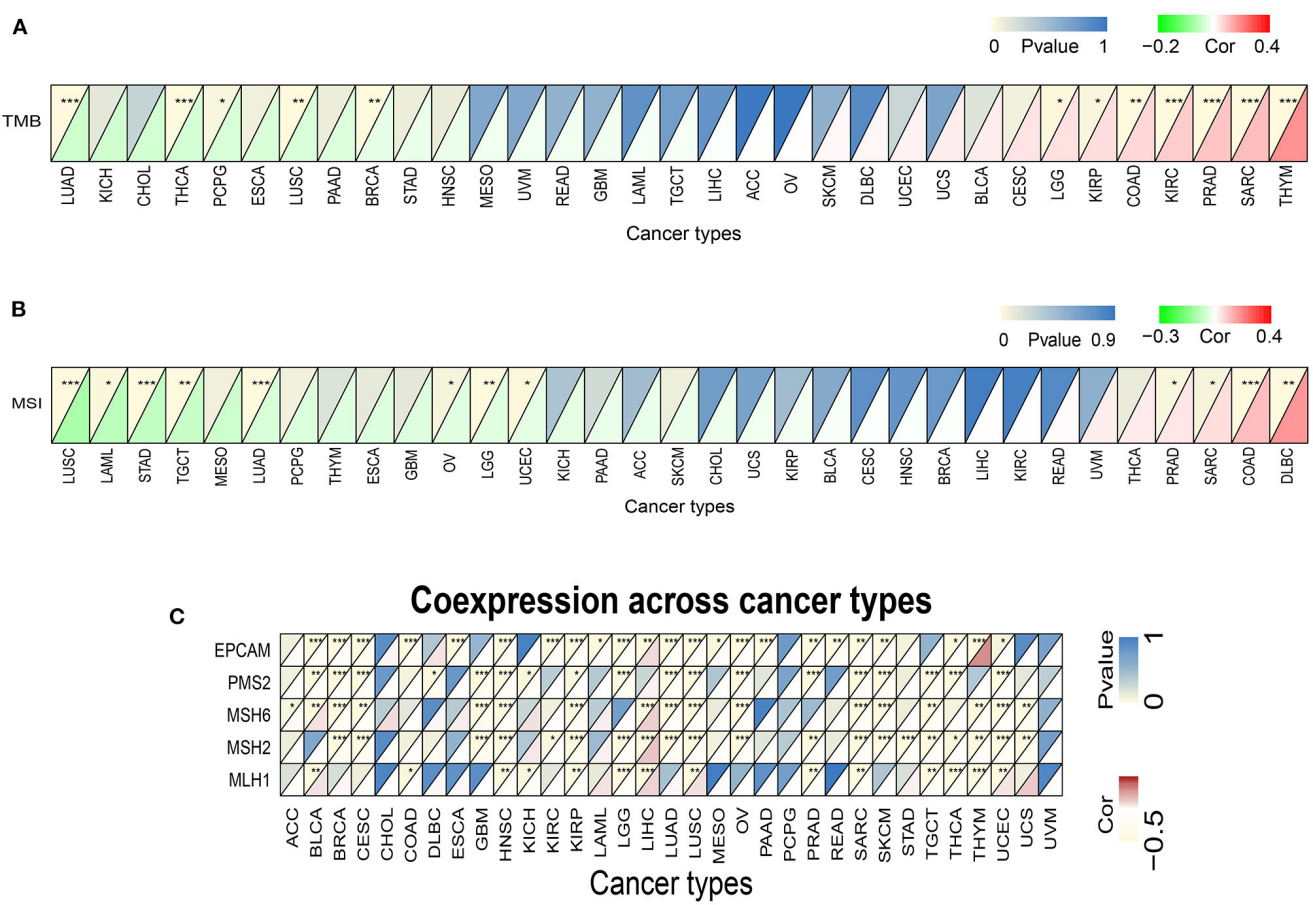
Associations between TREM2 expression and tumor mutational burden (TMB), microsatellite instability (MSI), and mismatch repair (MMR). **(A)** Heatmap illustrating the relationship between TREM2 and TMB. The top left triangle represents the P-value, and the bottom right triangle represents the correlation coefficient *p < 0.05, **p < 0.01, and***p < 0.001. **(B)** Heatmap illustrating the relationship between TREM2 and MSI. The top left triangle represents the P-value, and the bottom right triangle represents the correlation coefficient *p < 0.05, **p < 0.01, and ***p < 0.001. **(C)** Heatmap illustrating the association between TREM2 expression and MMR genes. For each pair, the top left triangle represents the P-value, and the bottom right triangle represents the correlation coefficient *p < 0.05, **p < 0.01, and ***p < 0.001.

Subsequently we investigated whether there were correlations between TREM2 expression levels and TMB and MSI, which both have essential connections with the sensitivity of immune checkpoint inhibitors. Hence, we studied the relationships between levels of MMR genes, including *MLH1, MSH2, MSH6, PMS2*, and *EPCAM*, and those of TREM2. The results demonstrated that, in 12 types of tumors, including breast cancer, colorectal cancer, lung cancer, glioma, and kidney cancer, TREM2 expression was related to TMB (Figure 8A). In another 12 types of tumor, including colorectal cancer, lung cancer, stomach cancer, and lymphoma, TREM2 expression was related to MSI (Figure 8B). Figure 8C illustrates the correlations between TREM2 expression levels and those of separate MMR genes. In most tumors, except for LIHC, MMR gene expression was clearly and significantly negatively correlated with TREM2 levels.

### Relationship Between TREM2 Expression and the Tumor Microenvironment

**Figure 9:**
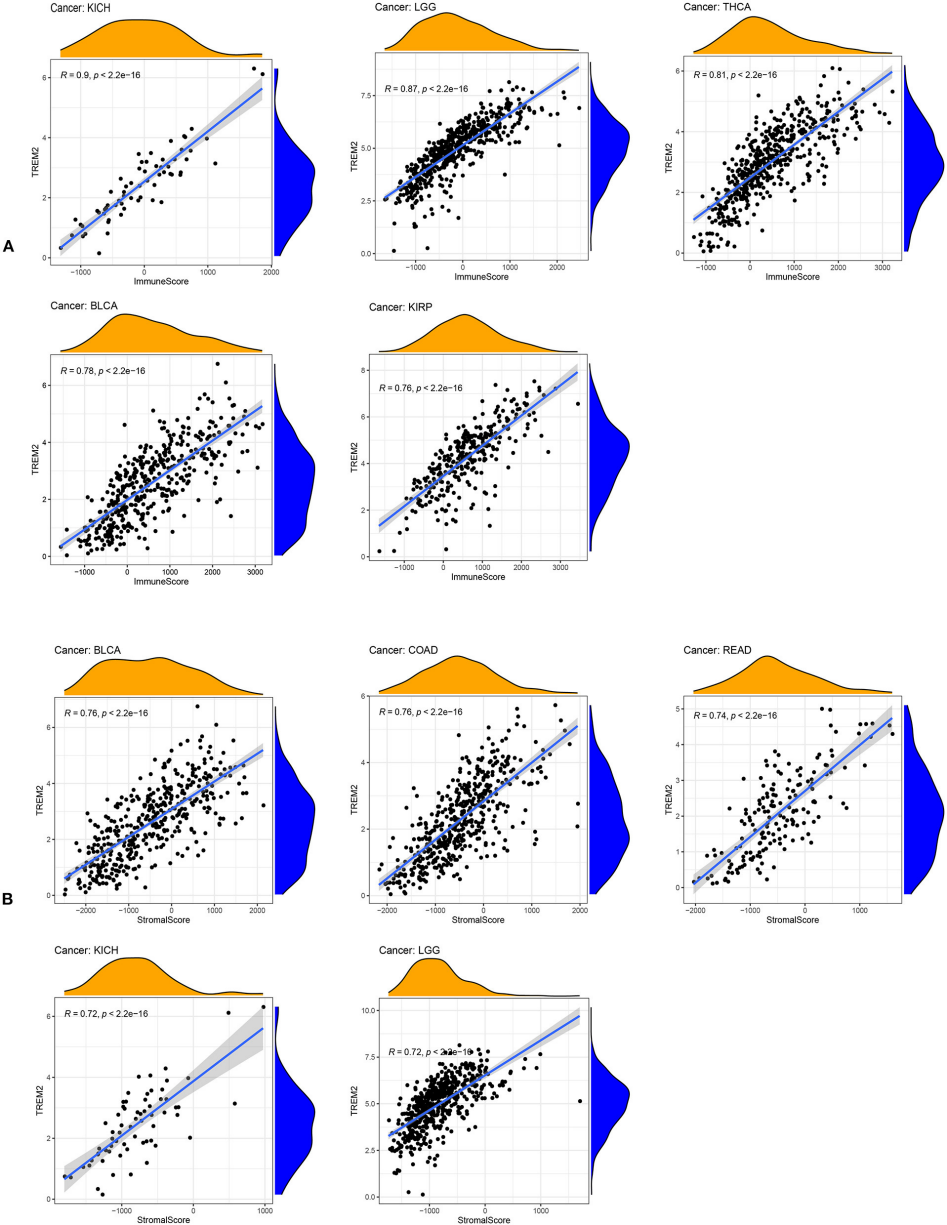
Five tumors with the highest correlation coefficients between TREM2 expression and the tumor microenvironment. **(A)** Correlation between TREM2 and immune scores in kidney chromophobe (KICH), brain lower grade glioma (LGG), thyroid carcinoma (THCA), bladder urothelial carcinoma (BLCA), and kidney renal papillary cell carcinoma (KIRP). **(B)** Correlation between TREM2 and stromal scores in BLCA, colon adenocarcinoma (COAD), rectum adenocarcinoma (READ), kidney chromophobe (KICH), and LGG.

An increasing number of reports indicate that the tumor immune microenvironment has a vital role in tumor occurrence and development (22, 23). Hence, it is important to further explore the pan-cancer relationship between TME and TREM2 expression. The ESTIMATE algorithm was used to calculate the stromal and immune cell scores in 33 types of cancer, and the relationships between TREM2 expression levels and these two scores analyzed. Our results reveal that, in DLBC, LAML, and THYM, TREM2 expression was significantly positively correlated with immune scores, as well as with stromal scores in pan-cancer analysis, except for in CHOL, DLBC, MESO, and LAML. The five tumors with the highest correlation coefficients are presented in Figure 9; the results for other cancers are shown in Supplementary Figures 2, 3.

### Relationship Between TREM2 Expression Levels and Levels of Tumor Immune Cell Infiltration

We next examined the relationship between TREM2 expression levels and the levels of infiltration of 26 immune-related cells. Our data demonstrate that levels of immune cell infiltration were significantly associated with TREM2 expression in most types of cancer (Supplementary Table 1). Six tumors, including BRCA (*n* = 18), CESC (*n* = 19), KIRC (*n* = 15), LUSC (*n* = 18), SKCM (*n* = 18), and STAD (*n* = 15), with the highest levels of correlation between TREM2 expression and degree of immune cell infiltration were screened for further analyses (Table 1). TREM2 expression levels were negatively correlated with levels of infiltrating memory B cells, naïve B cells, dendritic cells, eosinophils, lymphocytes, and NK cells in the six tumors analyzed. Further, TREM2 expression levels were correlated with multiple different subgroups of infiltrating macrophages. For example, TREM2 expression was negatively correlated with the levels of infiltrating M0 macrophages in BRCA and CESC, while it was positively associated with levels of infiltrating M1 macrophages, except in patients with BRCA. Similarly, there were positive correlations between TREM2 expression and levels of infiltrating M2 macrophages in these six tumors.

**Table 1:** Relationship between TREM2 expression and immune cell infiltration in different cancers.

**Cell type**	**BRCA**	**CESC**	**KIRC**	**LUSC**	**SKCM**	**STAD**
	**(P-value/Cor)**	**(P-value/Cor)**	**(P-value/Cor)**	**(P-value/Cor)**	**(P-value/Cor)**	**(P-value/Cor)**
Memory B cells	^***^/−0.11	^**^/−0.15	^***^/−0.19	^***^/−0.14	^***^/−0.18	^**^/−0.13
Naïve B cells	^***^/−0.12	−0.06	^*^/−0.11	−0.04	^**^/−0.13	^***^/−0.15
Dendritic cells	^***^/−0.09	^**^/−0.14	−0.08	^***^/−0.21	−0.04	−0.01
Activated dendritic cells	^***^/−0.23	^***^/−0.28	^***^/−0.19	^***^/−0.38	^***^/−0.2	^***^/−0.23
Resting dendritic cells	^*^/0.07	^***^/0.21	0.03	^***^/0.19	^*^/0.1	^***^/0.27
Eosinophils	^*^/−0.06	^***^/−0.24	^*^/−0.1	−0.03	^**^/−0.12	0.03
Lymphocytes	^***^/−0.12	^*^/−0.11	−0.04	^***^/−0.2	0.02	^***^/−0.26
Macrophages	^***^/0.11	^***^/0.33	^***^/0.17	^***^/0.31	^*^/0.09	^***^/0.35
M0 Macrophages	^***^/−0.18	^**^/−0.15	0.07	−0.06	−0.08	−0.06
M1 Macrophages	^**^/−0.07	^***^/0.38	0.05	^*^/0.11	^***^/0.18	^***^/0.16
M2 Macrophages	^***^/0.34	^***^/0.42	^***^/0.23	^***^/0.42	^***^/0.13	^***^/0.45
Mast cells	^*^/0.06	^***^/−0.21	^***^/−0.37	^***^/−0.15	^***^/−0.27	−0.07
Activated mast cells	0.03	^***^/−0.23	0.03	^***^/−0.18	^**^/−0.12	−0.1
Resting mast cells	0.05	0.06	^***^/−0.41	0.03	^***^/−0.22	0.04
Monocytes	^**^/0.07	−0.07	^***^/−0.21	0.07	0.04	0.01
Neutrophils	^***^/0.14	^***^/−0.27	0.03	0.04	−0.07	−0.01
Activated NK cells	−0.03	−0.03	−0.06	^***^/−0.13	^***^/0.21	−0.07
Resting NK cells	^**^/−0.08	^***^/−0.25	^***^/−0.34	^***^/−0.14	^***^/−0.25	−0.1
Plasma cells	^***^/−0.13	^***^/−0.28	^***^/−0.18	^***^/−0.22	−0.08	^***^/−0.25
Activated CD4 memory T cells	−0.04	^***^/0.19	0.06	^*^/0.1	^*^/0.1	^***^/0.15
Resting CD4 memory T cells	0.04	−0.11	^***^/−0.15	^***^/0.28	^***^/−0.14	^***^/−0.16
Naïve CD4 T cells	−0.05	^*^/−0.13	^***^/−0.29	^***^/−0.23	^***^/−0.2	^**^/−0.14
CD8T cells	0	^*^/0.12	^***^/0.19	−0.07	^***^/0.24	^**^/0.13
Follicular T helper cells	^***^/−0.15	0.06	0.08	^***^/−0.31	−0.07	^***^/−0.2
Gamma delta T cells	−0.05	0.06	0.08	0.07	0.07	0
Regulatory T cells	^***^/0.12	^***^/0.19	^***^/0.23	^***^/0.17	^***^/0.22	^**^/0.14

*BRCA, breast invasive carcinoma; CESC, cervical squamous cell carcinoma and endocervical adenocarcinoma; KIRC, kidney renal clear cell carcinoma; LUSC, lung squamous cell carcinoma; SKCM, skin cutaneous melanoma; STAD, stomach adenocarcinoma*.

* *p < 0.05*,

** p < 0.01, and

*** *p < 0.001*.

**Figure 10:**
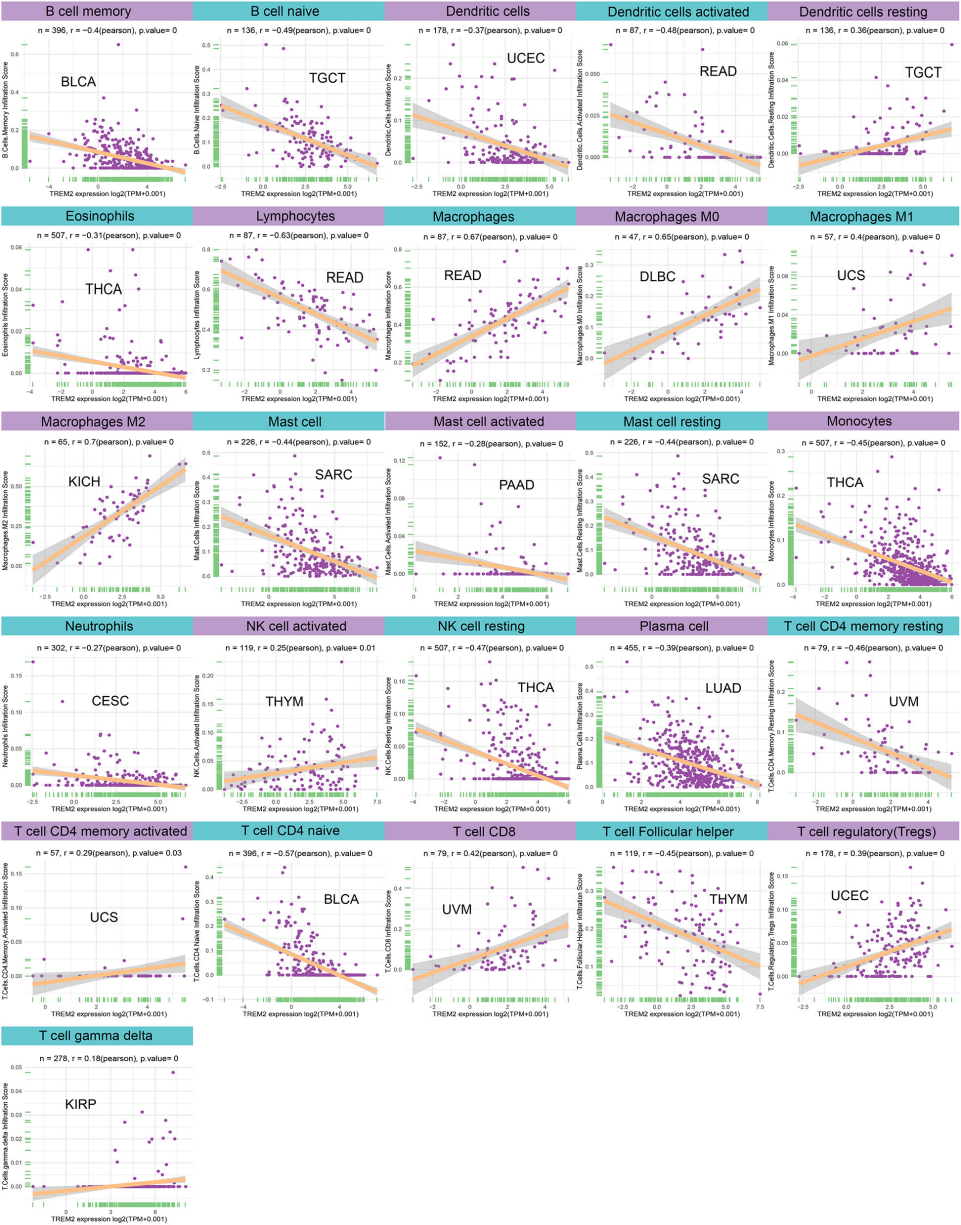
Relationship between TREM2 expression and tumor infiltration of different immune cells.

Moreover, diverse correlations were detected between TREM2 expression levels and different subsets of tumor infiltrating T cells. TREM2 expression was negatively correlated with the levels of infiltrating CD4 memory T cells (except in LUSC) and of infiltrating CD4 naïve T cells, and follicular helper T cells; however, it was positively correlated with levels of infiltrating CD8 and regulatory T cells (Tregs). Tumors with the highest correlation coefficients between the degree of infiltration and TREM2 expression for each type of immune cell are presented in Figure 10; data for other tumors are included in Supplementary Table 1.

**Figure 11:**
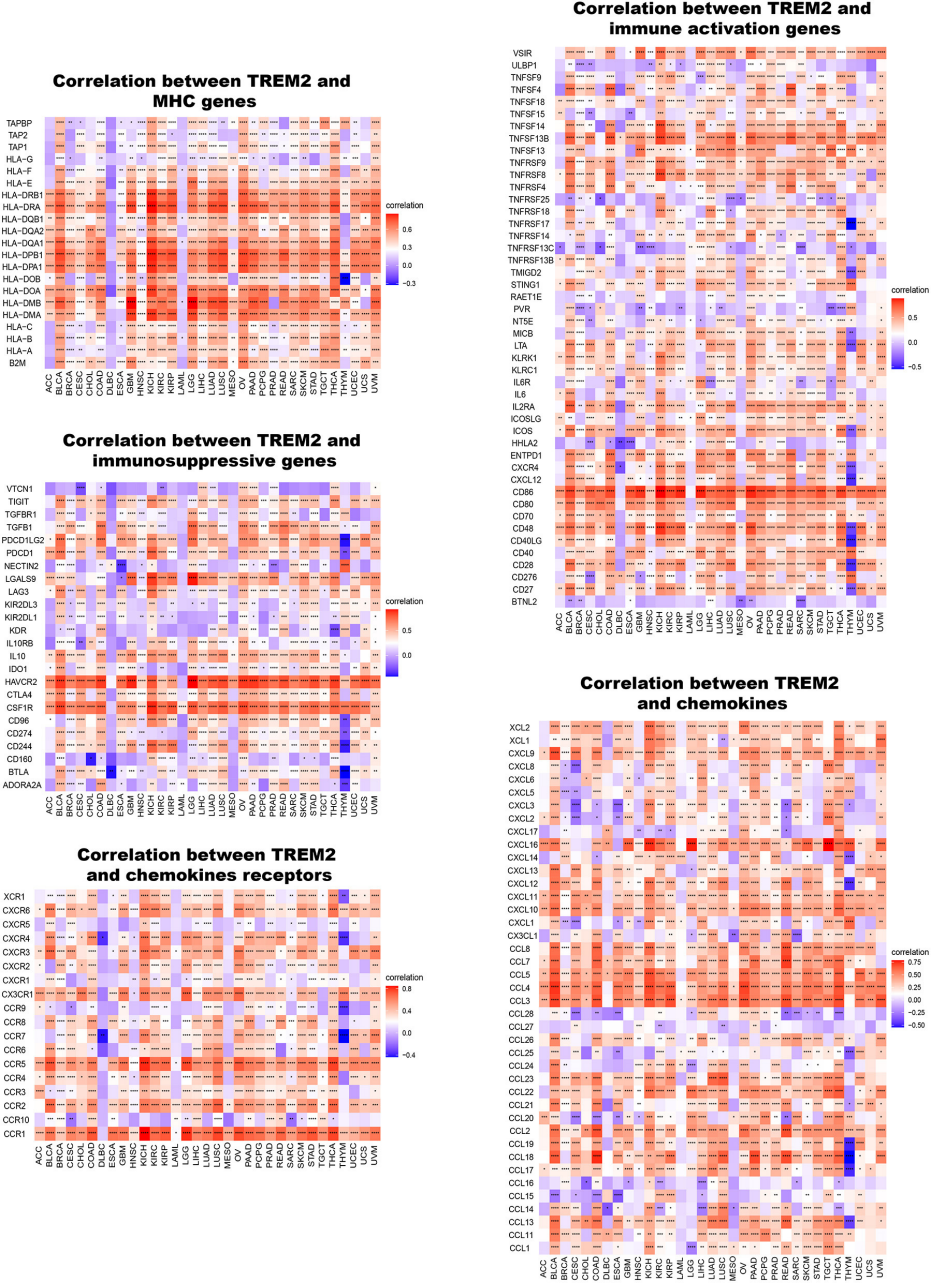
Co-expression of TREM2 and immune-related genes. *P < 0.05, **P < 0.01, ***P < 0.001, ****P < 0.0001.

Furthermore, we conducted gene co-expression analyses to explore the relationships between TREM2 expression and immune-related genes in 33 tumors. The analyzed genes encoded MHC, immune activation, immunosuppressive, chemokine, and chemokine receptor proteins. The resulting heatmap indicated that almost all immune-related genes were co-expressed with TREM2 (Figure 11) and the majority were positively correlated with TREM2 in all types of tumor, except DLBC and LAML (*p* < 0.05).

### Correlation of TREM2 Expression With DNA Methylation

**Figure 12:**
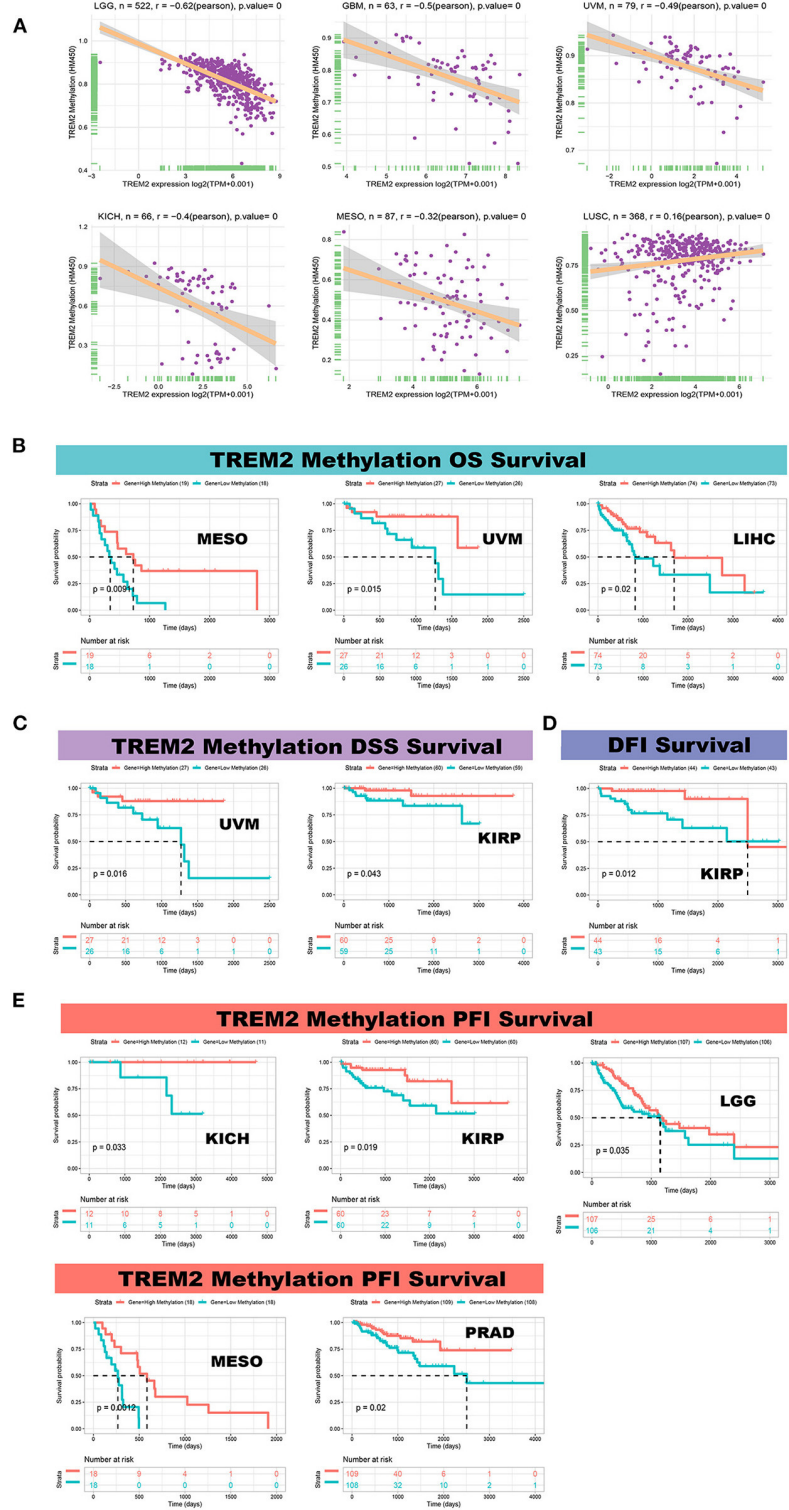
Correlation between TREM2 expression and gene promoter methylation. **(A)** Correlation between TREM2 expression and gene promoter methylation in brain lower grade glioma (LGG), glioblastoma multiforme (GBM), uveal melanoma (UVM), kidney chromophobe (KICH), mesothelioma (MESO), and lung squamous cell carcinoma (LUSC). **(B)** Correlation between TREM2 methylation overall survival in MESO, UVM, and liver hepatocellular carcinoma (LIHC). **(C)** Correlation between TREM2 methylation and disease-specific survival in UVM and kidney renal papillary cell carcinoma (KIRP). **(D)** Correlation between TREM2 methylation and disease-free interval in KIRP. **(E)** Correlation between TREM2 methylation and progression-free interval in KICH, kidney renal papillary cell carcinoma (KIPR), LGG, MESO, and prostate adenocarcinoma (PRAD).

We calculated the levels of correlation between TREM2 promoter methylation using the cBioPortal data set and identified significant correlations between gene expression and methylation in 20 tumors (Supplementary Figure 4). In STAD, LUAD, LUSC, and TGGT, there were negative correlations between TREM2 expression and promoter methylation levels. The five strongest positive correlations (LGG, GBM, uveal melanoma (UVM), KICH, and MESO) and one negative correlation (LUSC) are presented in Figure 12A. Further, we conducted Kaplan-Meier survival analysis to research the relationship between TREM2 promoter methylation and patient prognosis. TREM2 methylation level was a protective factor in patients with mesothelioma, uveal melanoma, and liver cancer, in terms of OS (Figure 12B). Regarding DSS, TREM2 methylation was a protective factor in patients with UVM and KIRP (Figure 12C), while TREM2 methylation level was only positively correlated with DFI in patients with KIRP (Figure 12D). Moreover, analysis of PFI data revealed an association between low TREM2 methylation level and poor prognosis in patients with KICH, kidney renal papillary cell carcinoma, LGG, MESO, and PRAD (Figure 12E).

### GSVA and GSEA

**Figure 13:**
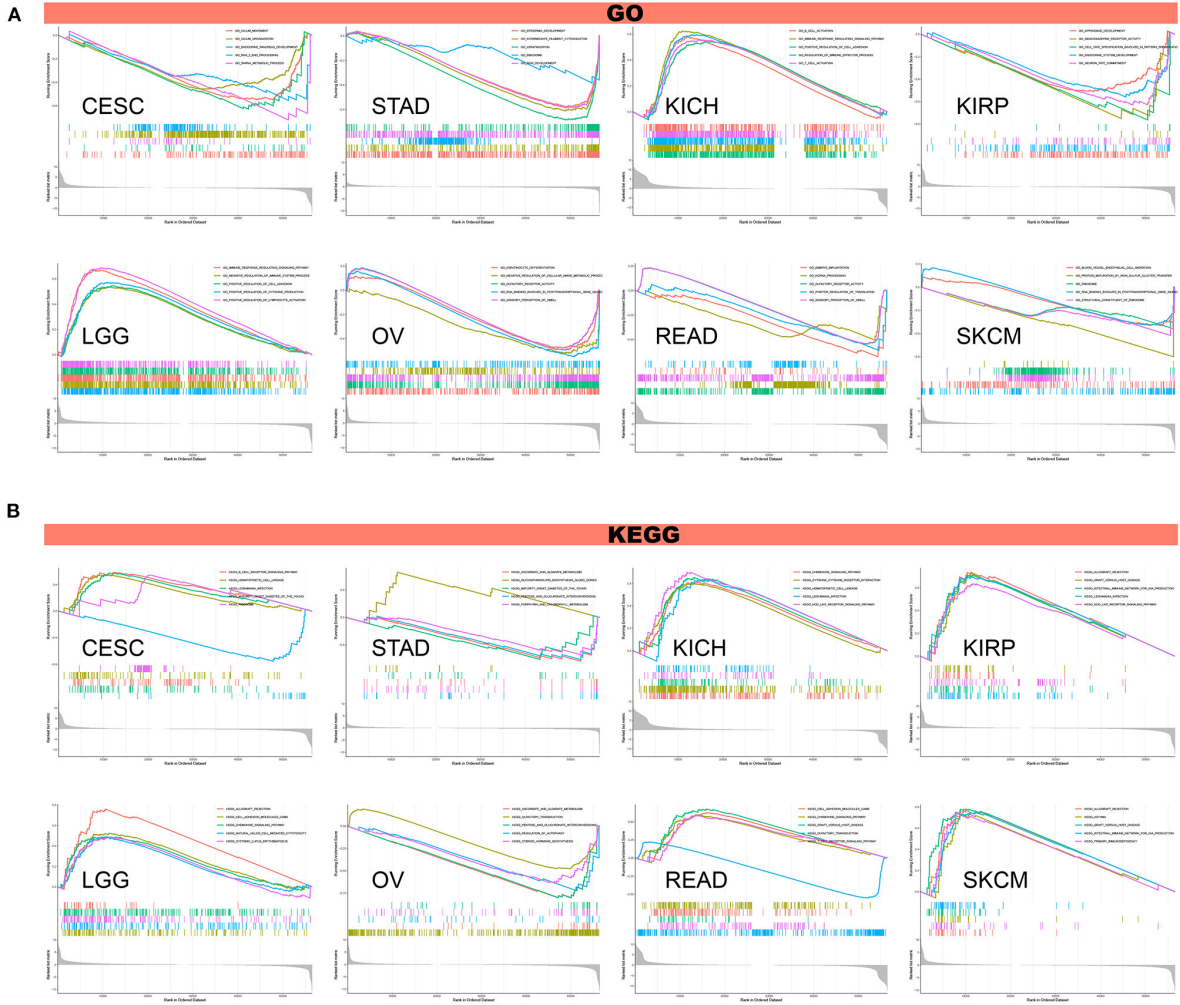
Results of GSEA. **(A)** GO functional annotation of TREM2 in various cancers. **(B)** KEGG pathway analysis of TREM2 in multiple cancers. Curves of different colors show different functions or pathways regulated in different cancers. Peaks on the upward curve indicate positive regulation and peaks on the downward curve indicate negative regulation.

To investigate the biological significance of TREM2 expression in different tumor tissues, we conducted GESA and GSVA. The results of GO functional annotation and KEGG pathway analysis are shown in Figure 13. The data indicate that TREM2 positively regulates cell adhesion and several immune-related functions in LGG and KICH, including B/T cell activation, immune responses, and immune regulation and signaling pathways. In contrast, TREM2 is predicted to be a negative regulator of the ribosome, RNA binding, snRNA, and other metabolic processes in CESC, STAD, KIRP, ovarian serous cystadenocarcinoma (OV), READ, and SKCM (Figure 13A). In CESC, KICH, KIRP, LGG, READ, and SKCM, TREM2 expression was positively correlated with hematopoietic cell lineage, *Leishmania* infection, chemokine signaling pathway, and some immune-related pathways, including allograft rejection, B/T cell receptor signaling pathway, natural killer cell-mediated cytotoxicity, and intestinal immune network for IgA production. Contrasting results were found for ascorbate and alternate metabolism and olfactory transduction in STAD and OV tumor cells (Figure 13B).

**Figure 14:**
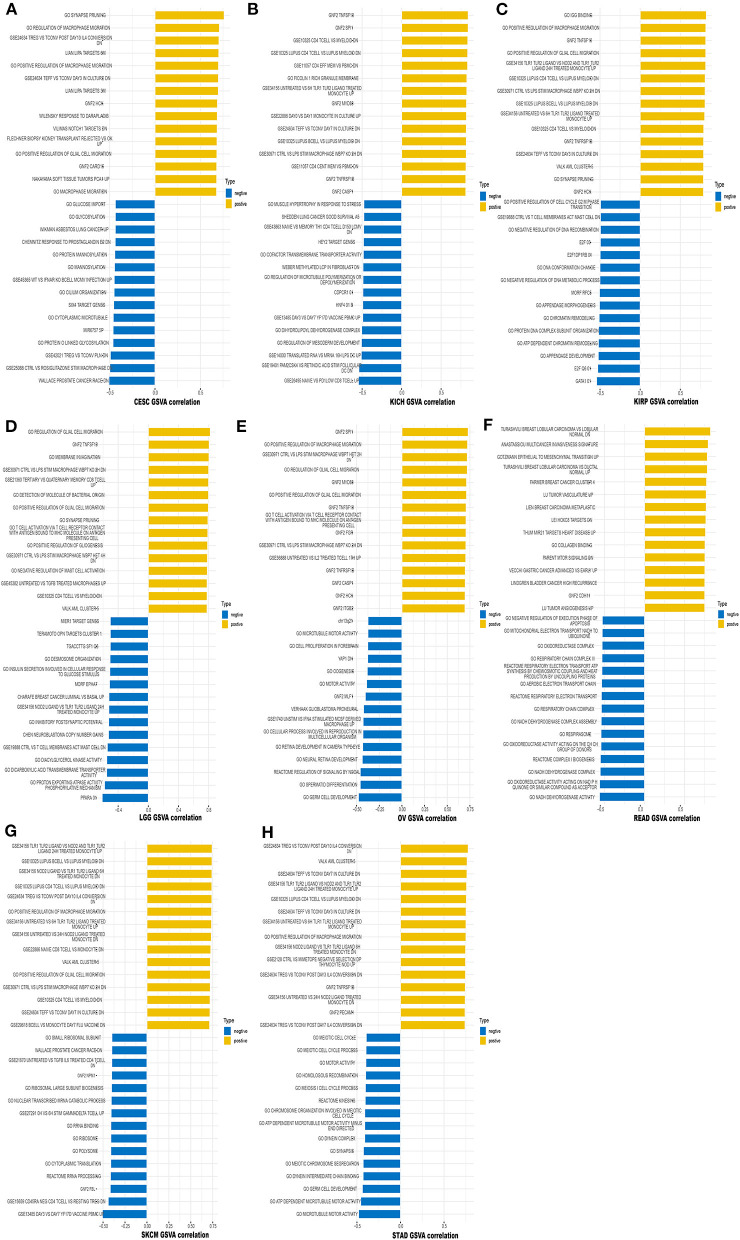
Results of GSVA. Yellow bars show the 15 pathways with the most significant positive correlation and blue bars show the 15 pathways with the most significant negative correlations. Horizontal axis represents the correlation coefficient. **(A)** Cervical squamous cell carcinoma and endocervical adenocarcinoma (CESC). **(B)** Kidney chromophobe (KICH). **(C)** Kidney renal papillary cell carcinoma (KIRP). **(D)** Brain lower grade glioma (LGG). **(E)** Ovarian serous cystadenocarcinoma (OV). **(F)** Rectum adenocarcinoma (READ). **(G)** Skin cutaneous melanoma (SKCM). **(H)** Stomach adenocarcinoma (STAD).

We also performed GSVA to further explore the biological significance of TREM2 expression in the above eight tumors. The top 15 pathways significantly positively and negatively associated with TREM2 expression in each tumor are presented in Figure 14. The results demonstrate that TREM2 expression is positively associated with several immune cell-related pathways, including B, CD4 T, and CD8 T cells, and immune factor-related pathways such as TNF, cell migration, and synaptic pruning. In contrast, TREM2 expression was negatively correlated with cell cycle-related pathways and specific metabolic pathways, such as glucose, glycosylation.

## Discussion

Our research shows that the TREM2 gene is highly expressed in 16 types of cancer, and IHC analysis confirms this tendency at the protein level. The results for glioma, gastric cancer, renal cancer, and liver cancer were similar to those of previous research (14, 15, 24, 25); however, Tang et al. (11) showed that TREM2 expression was decreased in hepatoma cells and most human HCC tissues, which contradicts our current results, possibly because more of the samples analyzed in our study were derived from tumors *in situ*, rather than metastases. Regarding colorectal carcinoma, our findings challenge those of previous research (12), which indicated that TREM2 is a potential prognostic biomarker, with expression downregulated in tumor tissues. This discrepancy may be due to differences in tumor samples, as the previous research included more highly proliferative colon cancers, with a focus on metastases. Interestingly, TREM2 expression levels in LUSC and LUAD were lower than those in normal tissues. Although the expression level of TREM2 was generally low in the entire tumor tissue, Yao et al. (26) reported that TREM2 expression was up-regulated on monocytes from patients with lung cancer compared with those from healthy individuals.

Our Kaplan-Meier survival analysis using TCGA data demonstrated that high TREM2 expression was linked to poor prognosis in LGG. Similarly, TREM2 expression was previously reported as associated with shorter survival time in patients with gastric cancer (14). Moreover, a recent study showed that TREM expression in peripheral blood mononuclear cells can serve as a prognostic indicator in patients with high-grade glioma (27). Our results also clarified that up-regulation of TREM2 expression is associated with poor prognosis in patients with renal cancer, and previous studies have demonstrated that TREM2 acts as an oncogene in the development of renal cell carcinoma (15). In contrast, high TREM2 expression is associated with good prognosis in patients with CESC, LUAD, and THCA. Regarding LIHC, our research reached the opposite conclusion from those of previous studies (25, 28).

In addition, we discovered that TREM2 expression is related to age in some types of cancer. TREM2 expression was lower in younger patients with LUAD, BRCA, PRAD, SARC, and THYM, while lower TREM2 expression was associated with older age in patients with CHOL. These results may have significance in guiding the choice of immunotherapy options in patients from different age groups. Our study also revealed that TREM2 expression was correlated with tumor stage in the majority of cancers, and was particularly different between stage I and II tumors. TREM2 expression on lung macrophages was previously reported to be positively correlated with pathological stage in lung cancer (13). These findings clearly demonstrate that TREM2 can be used as a biomarker to determine the prognosis of various cancers. Further, our study explored the relationship between TREM2 promoter methylation and cancer for the first time. We found that TREM2 expression was correlated with DNA methylation, and that TREM2 methylation level could serve as a biomarker of prognosis in patients with cancer.

TMB is a promising pan-cancer predictive biomarker (29) and can guide immunotherapy in the era of precision medicine (30). Previous research has shown that TMB can be used as a biomarker to improve immunotherapy efficacy in non-small-cell lung (31) and colorectal (32) cancers. Further, TMB can also predict prognosis after immunotherapy in pan-cancer patients (33). MSI is also an important biomarker in immune-checkpoint inhibitors (ICI) (32, 34). High-frequency MSI in colorectal cancer is an independent predictor of clinical characteristics and prognosis (35). Our study demonstrated that TREM2 expression is correlated with TMB in 12 cancer types and with MSI in 12 cancer types. This may indicate that the level of TMER2 expression will affect the TMB and MSI of cancer, thereby affecting the patient's response to immune checkpoint suppression therapy. This will provide a new reference for the prognosis of immunotherapy. We also found that, in most tumors, except for LIHC, TREM2 expression is negatively correlated with MMR gene expression. Based on existing research and our findings, we infer that tumors with high TREM2 expression, and high TMB and MSI may have a better prognosis after ICI treatment in cancers where TREM2 expression is positively correlated with TMB.

Our results show that TREM2 plays an essential role in cancer immunity. TME features serve as markers for evaluating tumor cell responses to immunotherapy and influence clinical outcomes (22). According to ESTIMATE scores, there were positive correlations between TREM2 expression and both stromal and immune cell content in the TME of 30 cancers. Tumor-infiltrating immune cells have important impacts on the occurrence and development of tumors and can antagonize or promote tumor occurrence and development (23). Previous research has reported that TREM2 expression functions in intracellular immunosuppression, and TREM2 expression can be induced in myeloid cells (36, 37). Xiong et al. (38) identified TREM2 overexpressing macrophage subpopulations and gamma delta T cell subpopulations in patients with melanoma who did not respond to immunotherapy. *Trem2* gene knockout model mice are more resistant to the growth of various cancers, and checkpoint immunotherapy can be improved by the TREM2 function of modifying tumor myeloid infiltrates (9). Our research further clarifies that TREM2 has a broader range of tumor applicability and confirms that TREM2 expression is closely involved in the biological processes of immune cells and immune-related molecules across most cancers. Further, our study also revealed the co-expression of TREM2 with genes encoding MHC, immune activation, immunosuppressive, chemokines, and chemokine receptor proteins. These results all indicate that expression of TREM2 is closely related to immune infiltration of tumor cells, affects patient prognosis, and proposes new targets for the development of immunosuppressants.

Furthermore, our enrichment analyses indicated that TREM2 can potentially impact cancer etiology or pathogenesis by functioning in cell adhesion; B/T cell activation, immune response, immune regulating, and signaling; RNA metabolism; and metabolic pathways. These data are consistent with previously published articles, indicating that expression of the TREM2 receptor signal on myeloid cells is regulated by the B cell activation linker and non-T cell activation linker proteins, and affects the macrophage inflammatory response (39) and activating Wnt/β-Catenin pathway (40).

In summary, our first pan-cancer analyses of TREM2 indicates that this factor is differentially expressed between tumor and normal tissues and reveals correlations of TREM2 expression with clinical prognosis and DNA methylation. Our findings suggest that TREM2 can be served as an independent prognostic factor of many tumors and for different tumors, the level of its expression level will bring different prognostic outcomes, which needs to be further studied for the specific role of TREM2 in each cancer. Moreover, TREM2 expression was associated with TMB, MSI, and immune cell infiltration across various cancer types. Its impact on tumor immunity also varies with tumor types. These findings may help to elucidate the role of TREM2 in tumorigenesis and development, and can provide a reference for the realization of more precise and personalized immunotherapy in the future.

## Data Availability Statement

Publicly available datasets were analyzed in this study. This data can be found here: <The RNA sequencing data, somatic mutation data, clinicopathological and survival data of 33 cancers were downloaded from UCSC Xena database (https://xena.ucsc.edu/). Tumor cell line's data were downloaded from the CCLE database (https://portals.broadinstitute.org/ccle/) TREM2 expression in 31 various tissues were downloaded from GTEx (https://commonfund.nih.gov/GTEx). Immunohistochemistry images of TREM2 protein expression were downloaded from the Human Protein Atlas (HPA) (http://www.proteinatlas.org/). The methylation HM450 data were downloaded from cBioPortal database (http://www.cbioportal.org/). All the datasets were open access datasets>.

## Author Contributions

WP conceived the study. XC and WP drafted the manuscript and performed the analysis. XW, KN, and YH performed the literature search and collected the data. LC and ZZ contributed to drafting the manuscript and interpreting data. All authors read and approved the final manuscript.

## Conflict of Interest

The authors declare that the research was conducted in the absence of any commercial or financial relationships that could be construed as a potential conflict of interest.
