# Supplementary figures and images for: Systematic Pan-Cancer Analysis Identifies TREM2 as an Immunological and Prognostic Biomarker
Source: Front Immunol. 2021 Feb 17;12:646523. doi: 10.3389/fimmu.2021.646523 (PMC7925850; doi:10.3389/fimmu.2021.646523)

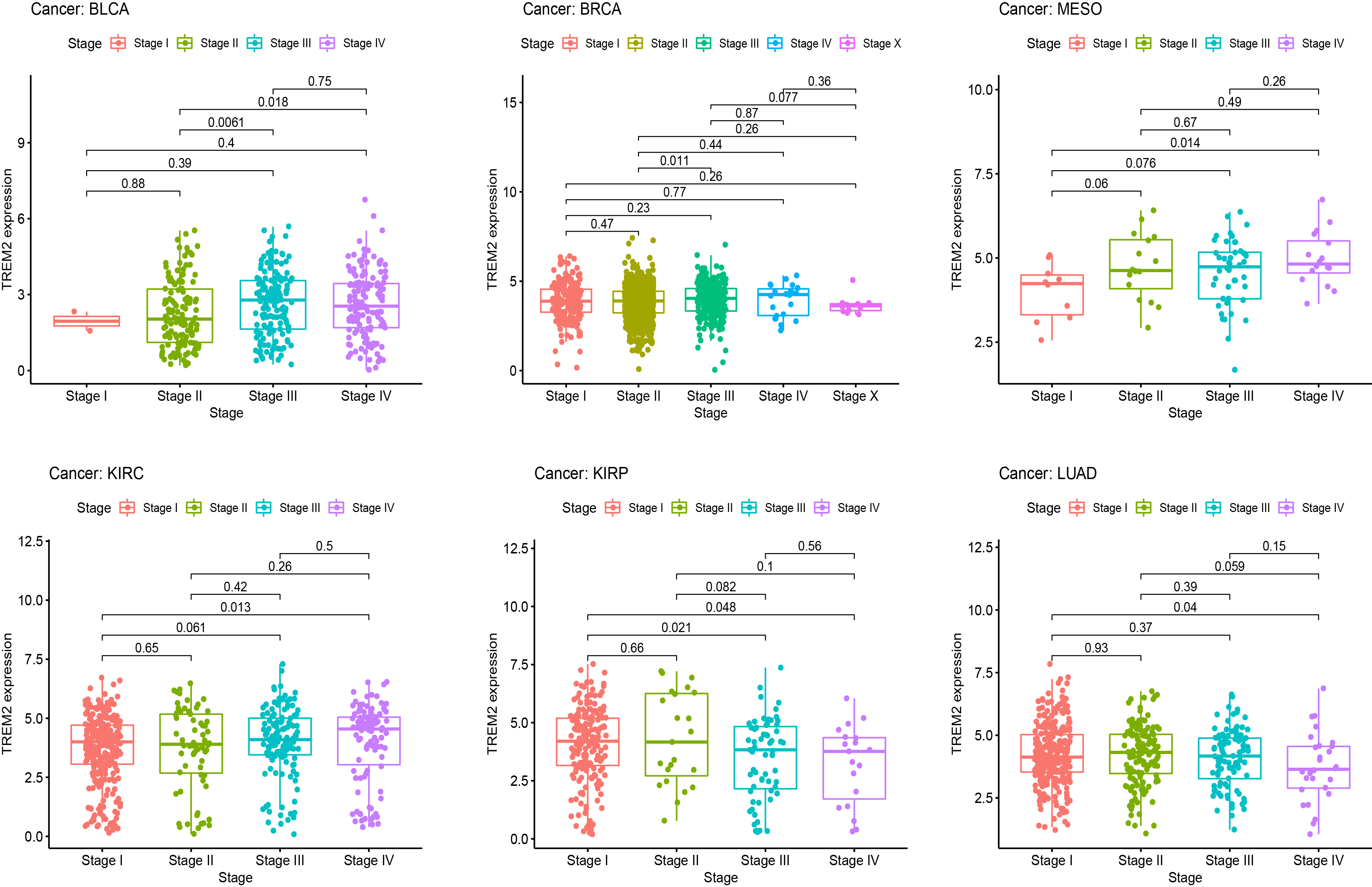

Supplement: Supplementary file 2 [file Image_1.JPEG]

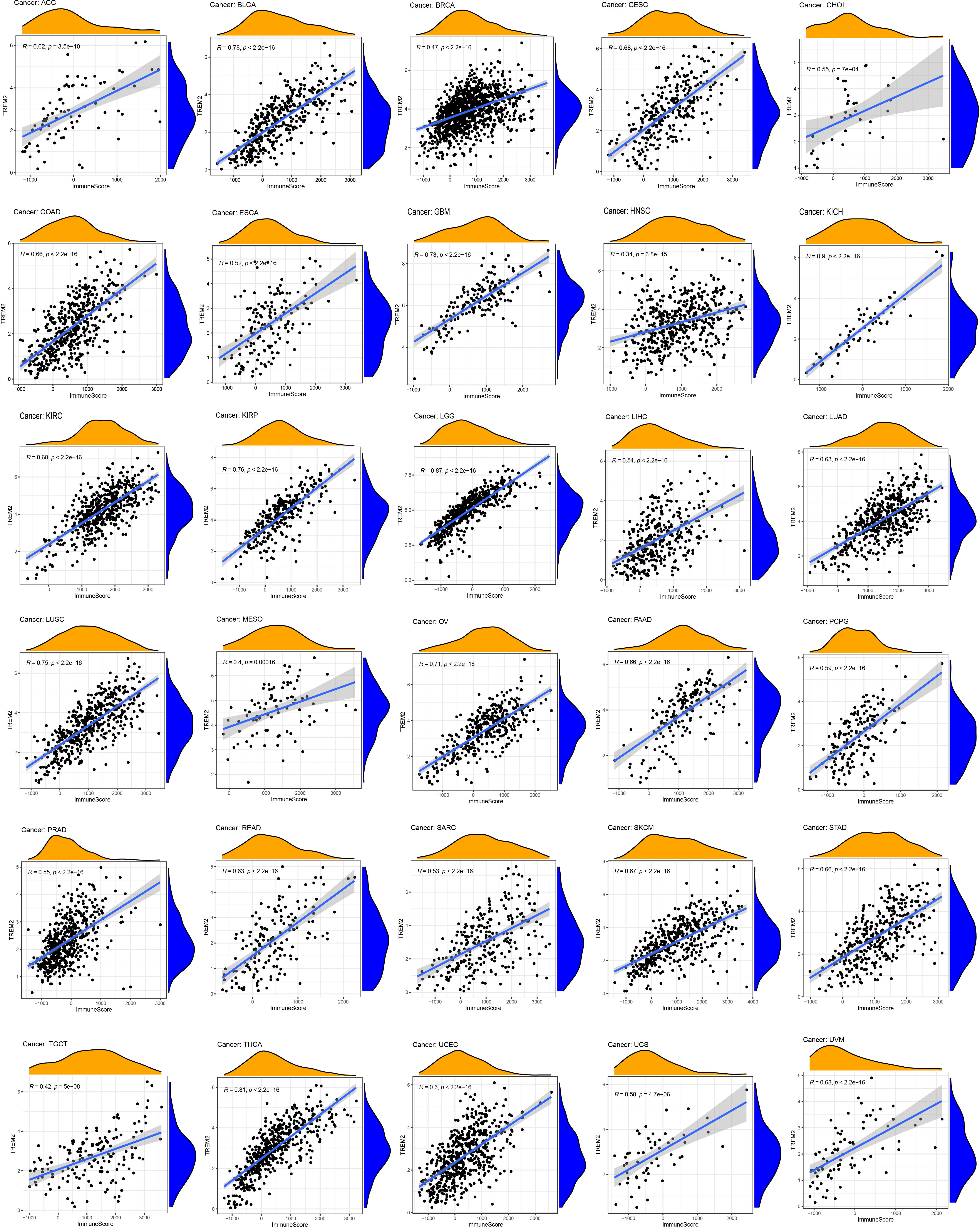

Supplement: Supplementary file 3 [file Image_2.JPEG]

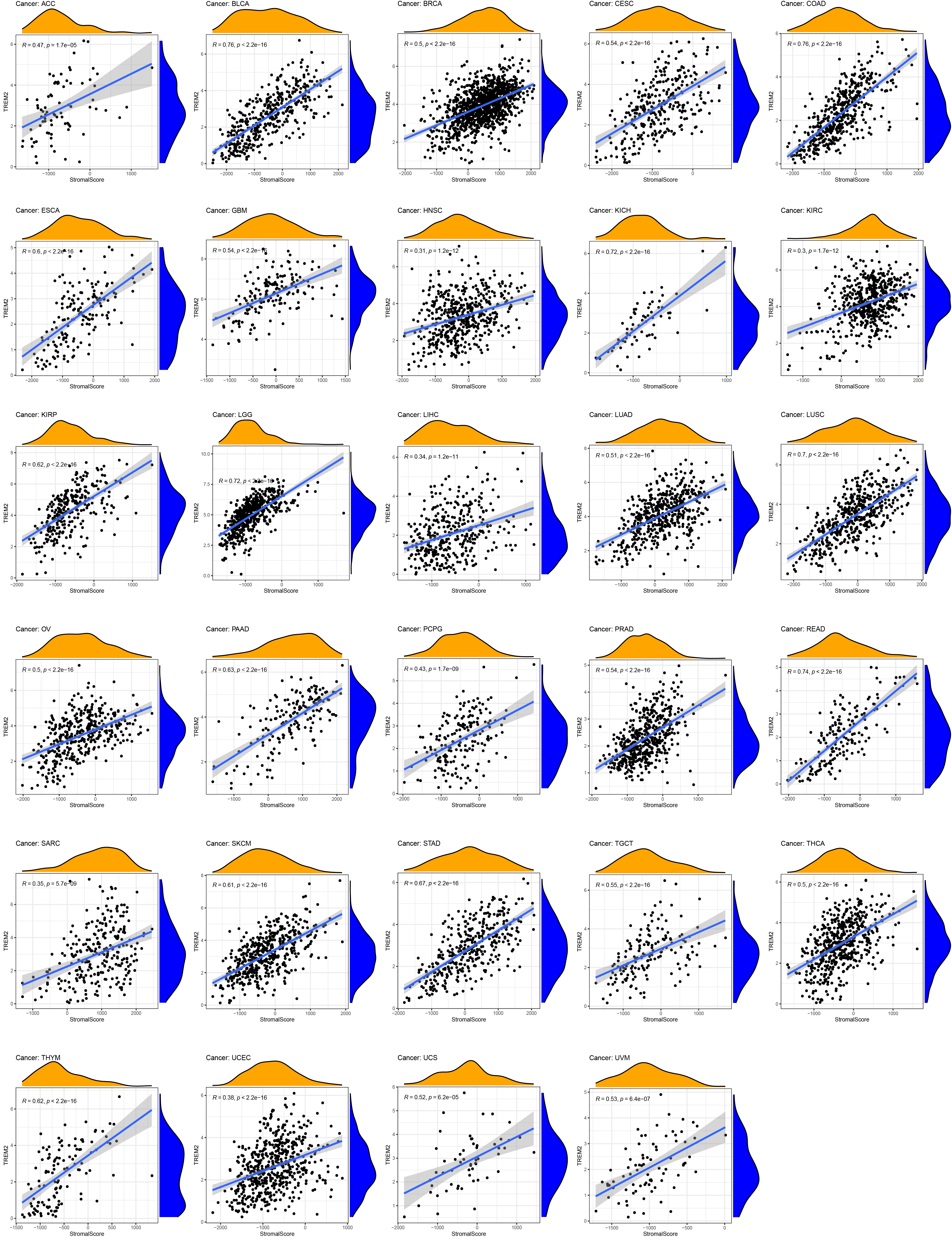

Supplement: Supplementary file 4 [file Image_3.JPEG]

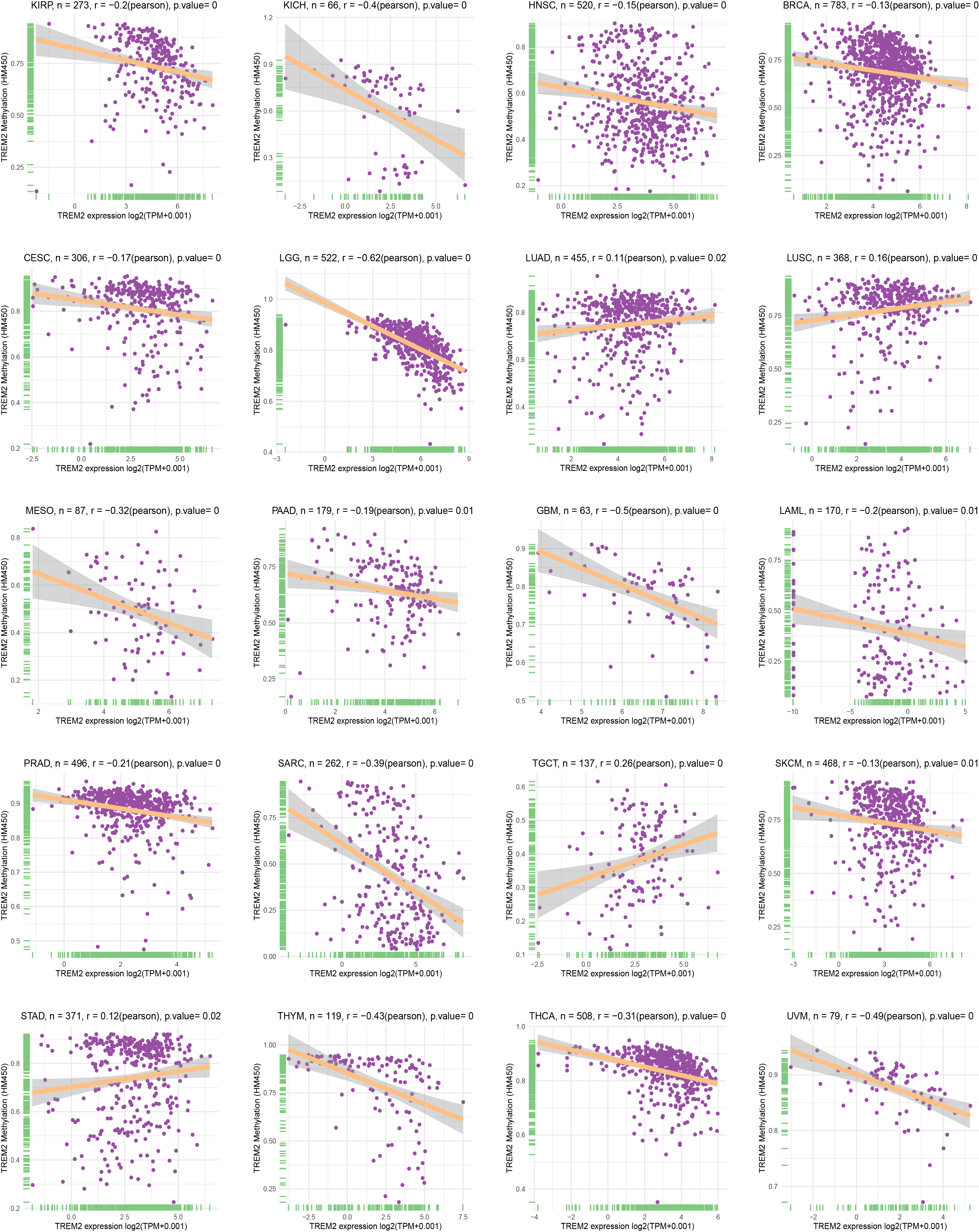

Supplement: Supplementary file 5 [file Image_4.JPEG]
